# Age-moderating effects and training characteristics of neuromuscular training for preventing knee injuries: a systematic review and meta-analysis

**DOI:** 10.3389/fpubh.2026.1822715

**Published:** 2026-08-28

**Authors:** Youwei Yao, Ming Pan, Xuesong Niu

**Affiliations:** 1 School of Strength and Conditioning, Shenyang Sport University, Shenyang, Liaoning, China; 2 Department of Pharmacy, Jinling Hospital, Affiliated Hospital of Medical School, Nanjing University, Nanjing, Jiangsu, China

**Keywords:** age, knee injury, meta-analysis, neuromuscular training, sports injury prevention

## Abstract

**Objective:**

Sports-related knee injuries are common and may cause pain, joint damage, reduced sport participation, and long-term physical activity limitations. Although neuromuscular training (NMT) is widely used for injury prevention, whether its effectiveness is moderated by age or intervention characteristics remains unclear. This systematic review and meta-analysis evaluated the effectiveness of NMT for preventing sports-related knee injuries and examined the influence of age and training characteristics.

**Methods:**

PubMed, Embase, Web of Science, The Cochrane Library, and EBSCO (SPORTDiscus) were searched from inception to September 30, 2025. Thirty studies were included following PRISMA guidelines. Random-effects meta-analysis, subgroup analyses, meta-regression, heterogeneity assessment, and risk-of-bias assessment using RoB 2 and ROBINS-I were performed.

**Results:**

Thirty studies were included. NMT significantly reduced knee injury risk in athletes (effect estimate = 0.670, 95% CI: 0.547–0.820, *p* < 0.001), with moderate heterogeneity (*I*^2^ = 58.5%). Age subgroup analyses showed protective effects in athletes aged >18 years (effect estimate = 0.566) and ≤18 years (effect estimate = 0.718), with no significant between-subgroup difference. Meta-regression did not identify study-level moderation by mean age or female proportion. Interventions incorporating landing-stabilization, plyometric, core, agility, or lower-limb strength training showed significant preventive effects, although between-subgroup differences for individual components were not significant. Multicomponent programs (≥3 components) were associated with significant risk reduction (effect estimate = 0.643), whereas programs with <3 components were not; the between-subgroup difference was significant (*p* = 0.045). Standardized programs significantly reduced injury risk (effect estimate = 0.592), whereas non-standardized programs did not. Dose-related analyses suggested that 15–20 min/session, 2–3 sessions/week, and durations >25 weeks may represent relatively stable and clinically interpretable patterns, although dose-related between-subgroup differences were not significant.

**Conclusion:**

NMT significantly reduces knee injury risk in athletes, with no clear evidence of age-related moderation. Structured, multicomponent, and standardized programs may have practical advantages, but findings on program structure, components, standardization, and dose should be interpreted cautiously because these analyses were exploratory.

**Systematic review registration:**

PROSPERO, under registration number CRD420261327352.

## 1 Introduction

Epidemiological studies have shown that injuries related to sports and recreational activities have become a major public health concern in many countries (1–4). Among all sports-related injuries, the knee is one of the most frequently affected and clinically consequential anatomical sites, with a high incidence in both competitive and recreationally active populations (5–8). Knee injuries not only interrupt training and competition, but are also often associated with long-term functional limitations and a substantially increased risk of early-onset osteoarthritis and chronic pain (9–13). Beyond these clinical consequences, knee injuries may also reduce athletes’ confidence, motivation to participate in sport, and long-term engagement in physical activity, thereby affecting both athletic development and lifelong health. In particular, in sports involving rapid deceleration, cutting, jumping and landing, and physical contact, anterior cruciate ligament (ACL) injuries, meniscal injuries, and patellofemoral joint-related disorders are especially common, imposing considerable long-term social and economic burdens (14, 15). In this context, the prevention of sports injuries is widely recognized as a major objective in the development of effective intervention strategies (16–18). Previous studies have indicated that the occurrence of lower-extremity injuries is closely associated with neuromuscular control, proprioceptive function, and movement patterns (14, 19). Deficits in neuromuscular control may lead to reduced joint stability, abnormal lower-limb alignment, and increased impact loading, thereby elevating the risk of knee injury during high-risk sporting activities (14, 20). On the basis of this theoretical framework, training interventions aimed at improving neuromuscular control have received increasing attention (21–23).

In recent years, numerous studies have evaluated the role of preventive interventions centered on neuromuscular training (NMT) in reducing the risk of sports injuries (24–28). NMT is a multifaceted training approach aimed at improving neuromuscular control. By enhancing sensory input and influencing central nervous system regulatory mechanisms, it strengthens unconscious motor responses and improves dynamic joint stability. Such programs typically integrate balance and proprioceptive training, lower-limb strength training, plyometric training, core training, agility and change-of-direction drills, and landing technique training, with the aim of improving dynamic postural control and joint stabilization capacity (23, 29). Multiple randomized controlled trials and prospective controlled studies have reported that structured NMT interventions can reduce the incidence of lower-extremity and knee injuries in athletes and have been implemented across a variety of team sports (16, 30–32). However, substantial variation exists across studies in terms of training content, intervention dose, and delivery mode, which complicates the interpretation of findings and their translation into practice. Specifically, existing studies differ markedly in the composition of intervention protocols. Some primarily employ balance or unstable-surface training, whereas others conceptualize NMT as a multicomponent intervention that incorporates several training elements (29, 33, 34). In addition, inconsistencies in methodological quality, sample characteristics, follow-up duration, and outcome definitions may further contribute to between-study heterogeneity (35–37).

Previous reviews have supported exercise-based and neuromuscular approaches to sports injury prevention, but their scope and analytical focus differ from those of the present review. Early reviews of sports injury prevention summarized a broad range of strategies, including bracing, insoles, proprioceptive training, strength training, and multicomponent exercise programs, and therefore provided limited knee-specific evidence on NMT itself (6, 35, 38). Other reviews focusing on lower-extremity injuries or youth sport populations suggested that balance, strength, plyometric, and movement-control exercises may reduce injury risk, but they often combined different anatomical injury outcomes or did not specifically examine sports-related knee injuries (37). In contrast, several more recent reviews have focused primarily on ACL injury prevention and have shown that neuromuscular and multicomponent training programs are effective, particularly among young and female athletes (39–41). However, ACL-specific evidence may not be directly generalizable to broader knee injury outcomes, which may include ligamentous, meniscal, patellofemoral, and other knee-related injuries with different mechanisms. Moreover, the potential moderating role of age, as well as program-level factors such as program composition, standardization, and dose, have not been consistently examined together in knee-injury-specific meta-analyses. From a practical perspective, clarifying the role of age remains important because athletes of different age groups may differ in neuromuscular maturation, tissue adaptability, and training-related learning effects, which may theoretically influence their response to NMT. However, existing evidence remains insufficient to determine whether age modifies the effectiveness of NMT for broader sports-related knee injury outcomes.

Therefore, the present study conducted a systematic review and meta-analysis to synthesize evidence from randomized controlled trials and prospective controlled studies, with the aim of evaluating the overall effectiveness of NMT in preventing sports-related knee injuries. In addition, this study further examined the relationships between intervention effects and age group, as well as key characteristics of training protocols. The findings are expected to provide evidence-based support for the scientific design and implementation of sports injury prevention programs and to inform the development of knee injury prevention strategies for diverse groups of athletes.

## 2 Methods

### 2.1 Registration

This systematic review and meta-analysis was conducted in accordance with the Preferred Reporting Items for Systematic Reviews and Meta-Analyses (PRISMA) statement. The study protocol was prospectively registered in the International Prospective Register of Systematic Reviews (PROSPERO) under registration number CRD420261327352.

### 2.2 Literature search strategy

A systematic literature search was conducted in PubMed, Embase, Web of Science, the Cochrane Library, and EBSCO (SPORTDiscus) from database inception to September 30, 2025. The search strategy combined keywords and Medical Subject Headings (MeSH) terms, including but not limited to knee, neuromuscular training, prevention, training, and injury. The detailed search strategies for each database are provided in Supplementary Appendix 1.

### 2.3 Eligibility criteria

The eligibility criteria were established *a priori* according to the PICOS framework and are summarized in Table 1. Eligible studies were required to include physically active participants engaged in sports or organized physical activity, evaluate neuromuscular training interventions aimed at preventing sports-related knee injuries, include a control or comparison group, report knee injury incidence or sufficient data for effect-size calculation, and use a prospective controlled design.

**Table 1:** PICOS-based inclusion and exclusion criteria.

PICOS domain	Inclusion criteria	Exclusion criteria
Population	Athletes or physically active individuals participating in organized sports or sport-related training, without restrictions on sex, age, competitive level, or sport type.	Non-sporting general populations, clinical patient populations, postoperative rehabilitation populations, or participants with pre-existing knee injuries or diagnosed neurological or musculoskeletal diseases unrelated to sports injury prevention.
Intervention	Neuromuscular training or exercise-based prevention programs including one or more components such as strength training, balance/proprioceptive training, plyometric training, landing technique training, agility/change-of-direction drills, core training, or movement-control training.	Interventions not primarily based on neuromuscular or exercise-based training, such as bracing, taping, insoles, education-only programs, surgical or pharmacological interventions, or rehabilitation programs after injury.
Comparator	Usual training, routine warm-up, no specific preventive intervention, or other control conditions without structured NMT.	Studies without a control or comparison group.
Outcomes	Sports-related knee injury incidence, knee injury events, injured participants, incidence rates, or effect estimates allowing calculation or extraction of relative injury risk.	Studies not reporting knee-specific injury outcomes, studies reporting only biomechanical, performance, strength, balance, or adherence outcomes without injury incidence, or studies with insufficient data for effect-size calculation after attempts to obtain missing information.
Study design	Prospective controlled studies, including randomized controlled trials, cluster-randomized trials, quasi-experimental studies, and prospective cohort studies with a control group.	Reviews, meta-analyses, editorials, commentaries, case reports, cross-sectional studies, retrospective studies, animal studies, laboratory-only studies, conference abstracts without sufficient data, duplicate publications, non-English articles, or full texts unavailable.

### 2.4 Data extraction

Data extraction was independently performed by two reviewers for all included studies. Any disagreements were resolved through discussion, and, when necessary, adjudicated by a third reviewer. The extracted information included general study characteristics, participant characteristics, intervention characteristics, and outcome measures. General study characteristics included the first author, year of publication, country, and study design. Participant characteristics included sample size, sex composition (proportion of female participants), age information (mean, range, or median), sport type, and competitive level. For studies with missing information on age, outcome data, or key methodological characteristics, the corresponding authors were contacted by email to request additional data.

For outcome data extraction, knee injury outcome data were extracted according to the reporting format used in each original study. For studies reporting count-based outcomes, the number of knee injury events or injured participants and the corresponding group sample sizes were extracted for the intervention and control groups. When available, exposure information was also extracted and retained, including athlete-exposures, player-hours, training or match exposure time, person-time, follow-up duration, and incidence rates. We also recorded whether each study reported first knee injuries only, total knee injury events, recurrent injuries, or whether this distinction was unclear. When studies explicitly reported first-injury data, first knee injuries were preferentially extracted. When studies reported total knee injury events or incidence rates without clearly distinguishing first and recurrent injuries, the reported total events or rate-based outcomes were used as appropriate, and the injury-counting approach was recorded for interpretation. If the original article did not clearly state whether recurrent injuries were included, this was coded as unclear. Detailed information on injury definitions, surveillance characteristics, exposure denominators, and recurrent-injury handling is provided in Table S2 of Supplementary Appendix 2.

With regard to age- and sex-related variables, age information was systematically extracted for all study populations. These data were used to perform age-stratified subgroup analyses (≤18 years vs. > 18 years) and meta-regression analyses using the study-level mean age as a continuous variable. In addition, the proportion of female participants was extracted as a continuous covariate for meta-regression analysis.

Regarding intervention characteristics, the specific components and implementation features of neuromuscular training (NMT) were systematically extracted, including training content, delivery mode, whether the intervention was supervised, and the training conditions in the control group. To support the prespecified subgroup analyses of training characteristics, intervention features were coded and categorized as follows. (1) Training component coding: Based on explicit descriptions of intervention content in the original articles, two reviewers independently coded each study to determine whether the intervention included the following training component categories: landing stabilization training, plyometric training, core training, agility training, lower-limb strength training, stretching training, and balance training. A component was classified as “present” only when the study explicitly described the corresponding training unit (e.g., exercise, module, or training segment). If the article did not mention the component or if its presence could not be clearly determined, it was classified as “absent.” Based on these coding results, subgroup analyses were conducted for each training component separately using a present vs. absent comparison. (2) Number of training components: Based on the above coding, the number of training component categories included in each study was counted and classified according to the prespecified threshold into ≥3 components and <3 components for subgroup analysis. To avoid duplicate weighting, all subgroup analyses ensured that each study contributed only one effect size. (3) Standardization of training protocols: To compare standardized versus non-standardized NMT programs, each study was assessed against five prespecified criteria: (a) a fixed and clearly defined training structure; (b) a clearly specified training dose; (c) clearly defined progression or difficulty levels; (d) explicit quality-of-execution or technical standards; and (e) sufficient detail to allow protocol reproducibility. These criteria were not derived from a previously validated scoring instrument. Rather, they were developed *a priori* by the review team and were informed by features commonly emphasized in previous neuromuscular training and injury-prevention literature, including structured training content, dose specification, progressive difficulty, technical execution quality, and reproducibility of the intervention protocol (23, 42–45). Studies meeting ≥4 criteria were classified as standardized NMT, whereas those meeting ≤3 criteria were classified as non-standardized NMT. This threshold was prespecified to distinguish programs with most key standardization features from those with incomplete or insufficiently described protocol characteristics. Classification was performed independently by two reviewers, with disagreements resolved through discussion or adjudication by a third reviewer. Study-level judgments for each standardization criterion and the final standardized/non-standardized classification are presented in Supplementary Appendix 2, Table S5.

With regard to training dose, dose-related variables were systematically extracted, including session duration, weekly training frequency, and total intervention duration, to support subsequent dose-related subgroup analyses. To standardize reporting and facilitate quantitative comparison, all dose data were converted into continuous variables and expressed as minutes per session, sessions per week, and weeks, respectively, and were then categorized according to prespecified thresholds for subgroup comparisons. When fixed values were directly reported, the original data were used. When dose information was reported as ranges (e.g., 10–15 min, 2–3 sessions/week), the midpoint of the range was used as an approximation. For data reported using ≥ or ≤ symbols, or as approximate values, the threshold or reported value was taken as the point estimate (e.g., ≥2 sessions/week was coded as 2 sessions/week). If training frequency or session duration varied across intervention phases, a weighted average based on the duration (in weeks) of each phase was calculated to obtain the study-level mean dose. If a single training session consisted of multiple segments, the durations of all segments were summed to determine the total session duration. Total intervention duration was uniformly converted into weeks: studies reporting duration in days, months, or explicit start and end dates were converted using days ÷ 7, months × 4.345, or the number of days between the start and end dates ÷ 7, respectively, where 4.345 represents the average conversion factor from months to weeks (4.345 = [365/12]/7). For studies describing the intervention duration by season, duration was likewise converted into weeks when clear start and end time information was available. If the necessary information was insufficient for conversion, the relevant dose variable was recorded as missing and excluded from the corresponding dose-covariate analysis. Because these conversions involved approximations, the derived dose variables were used only for exploratory subgroup analyses and should be interpreted as study-level estimates rather than exact measures of the dose actually completed by individual participants.

### 2.5 Study selection process

The study selection process followed the PRISMA 2020 statement. After all retrieved records were imported into EndNote and duplicates were removed, two reviewers independently conducted the initial screening of titles and abstracts, followed by full-text assessment, in accordance with the predefined inclusion and exclusion criteria. Inter-reviewer agreement was calculated separately for title/abstract screening and full-text eligibility assessment using Cohen’s kappa coefficient before consensus discussion. Any disagreements were resolved through discussion or, when necessary, by consultation with a third reviewer.

### 2.6 Risk-of-bias assessment

Risk of bias was assessed according to study design. Randomized controlled trials were assessed using the revised Cochrane Risk of Bias tool for randomized trials (RoB 2). The assessed domains included bias arising from the randomization process, bias due to deviations from intended interventions, bias due to missing outcome data, bias in measurement of the outcome, and bias in selection of the reported result. For cluster-randomized trials, the additional RoB 2 domain concerning the timing of identification and recruitment of participants within clusters was considered where applicable. Each domain and the overall judgment were rated as low risk of bias, some concerns, or high risk of bias. Non-randomized controlled intervention studies were assessed using the Risk Of Bias In Non-randomized Studies of Interventions tool (ROBINS-I). The assessed domains included bias due to confounding, bias in selection of participants into the study, bias in classification of interventions, bias due to deviations from intended interventions, bias due to missing data, bias in measurement of outcomes, and bias in selection of the reported result. Each domain and the overall judgment were rated as low, moderate, serious, critical, or no information. The overall judgment was not calculated as a total score, but was determined according to the ROBINS-I guidance, taking into account the most serious domain-level judgment. Risk-of-bias assessment was independently performed by two reviewers. Inter-reviewer agreement for risk-of-bias judgments was calculated using Cohen’s kappa coefficient before consensus discussion. Disagreements were resolved through discussion, and unresolved discrepancies were adjudicated by a third reviewer.

### 2.7 Statistical analysis

All statistical analyses were performed using Comprehensive Meta-Analysis (CMA), version 3.0. Effect estimates were harmonized on the natural logarithmic scale and synthesized using a generic inverse-variance random-effects approach. For studies reporting knee-injury counts and total group sample sizes, event-count-based odds ratios (ORs) were calculated from the corresponding 2 × 2 data. For studies reporting adjusted risk ratios (RRs), incidence rate ratios (IRRs), rate ratios, or other exposure-adjusted relative effect estimates with corresponding 95% confidence intervals (95% CIs), the reported estimates were extracted to preserve the original adjustment for clustering, exposure time, or study design and were transformed to their natural logarithms for pooling. Because exact mathematical conversion of an RR or IRR to an OR requires additional information, such as baseline risk, exposure time, or the full event-count structure, which was not consistently available across studies, the reported RRs, IRRs, and rate ratios were pooled as log-transformed relative effect estimates rather than being mathematically converted into exact ORs.

For the event-count-based analyses, the OR was calculated as:


$$\text{OR}=\frac{a/b}{c/d}=\frac{\mathrm{ad}}{\mathrm{bc}}$$

Where a and c represent the reported knee-injury counts in the intervention and control groups, respectively, and b and d represent the corresponding non-event counts, calculated by subtracting the reported knee-injury counts from the total group sample sizes.

The log-transformed effect estimate and its standard error were calculated as follows:


$$\hat{\theta }=\ln(\text{OR})$$

$$\mathrm{SE}(\hat{\theta })=\sqrt{\frac{1}{a}+\frac{1}{b}+\frac{1}{c}+\frac{1}{d}}$$

When a study reported an adjusted RR, IRR, rate ratio, or other relative effect estimate with a 95% CI, the reported estimate was transformed as:


$$\hat{\theta }=\ln(\text{reported relative effect estimate})$$

and the standard error was calculated as:


$$\mathrm{SE}(\hat{\theta })=\frac{\ln(\text{upper}\;95\%\mathrm{CI})-\ln(\text{lower}\;95\%\mathrm{CI})}{3.92}$$

When zero events occurred in one cell of a 2 × 2 table, a continuity correction of 0.5 was added to each cell before calculating the effect estimate. Unless otherwise specified, all statistical tests were two-tailed, and the threshold for statistical significance was set at *p* < 0.05. To account for within-study sampling variance and between-study heterogeneity, the primary meta-analysis was conducted using a generic inverse-variance random-effects model, with between-study variance estimated using the DerSimonian–Laird method. For ease of interpretation, pooled logarithmic estimates were back-transformed and presented as relative effect estimates.

The generic inverse-variance approach is consistent with methodological guidance provided in the Cochrane Handbook for Systematic Reviews of Interventions, which recommends analysing ratio measures, including ORs, RRs, rate ratios, and hazard ratios, on the natural logarithmic scale and synthesizing study-specific effect estimates with their corresponding standard errors when appropriate (46, 47). Because the included effect measures represented related but non-identical estimands, the primary pooled estimate was interpreted as a pooled log-transformed relative effect, and its back-transformed value as a summary relative effect across different ratio measures rather than as a conventional pooled OR, RR, or IRR. The study-specific effect estimates used in the primary meta-analysis are summarized in Supplementary Appendix 2, Tables S3, S4.

Previous meta-analyses have synthesized evidence reported using different ratio measures when the included studies addressed a common exposure or intervention contrast and a sufficiently comparable outcome domain (48, 49). Generic inverse-variance methods have also been used in intervention reviews to analyse reported risk- and rate-based effect estimates (50). In the present review, all included studies compared NMT with a control condition, reported sports-related knee-injury outcomes, and used ratio measures with a common null value of 1 and a consistent direction of benefit. Nevertheless, risk-based and rate-based estimates were not assumed to represent identical or interchangeable estimands. Accordingly, sensitivity analyses stratified by effect-estimate type were conducted to assess whether the principal conclusions were influenced by combining risk-based and rate-based estimates.

#### 2.7.1 Primary analysis and subgroup analyses

The primary analysis was based on the incidence data for knee injuries from all included studies to calculate the overall pooled effect size. Prespecified subgroup analyses were then performed using a mixed-effects model according to the following categories: (1) Age group: ≤18 years and >18 years; (2) Sex-specific age subgroup analyses: where data permitted, the same age-based subgroup analyses were conducted separately for male and female samples; (3) Intervention characteristics: subgroup analyses were conducted according to the presence or absence of specific training components, the number of training components (≥3 vs. < 3), the degree of training standardization (standardized vs. non-standardized), and training dose characteristics (session duration, weekly training frequency, and total intervention duration).

#### 2.7.2 Random-effects meta-regression

To examine whether age and sex composition moderated the effect of NMT on the prevention of knee injuries, random-effects meta-regression analyses were performed. In these analyses, the study-level mean age or the proportion of female participants was entered as a continuous independent variable, and the log-transformed relative effect estimate was used as the dependent variable. Two models were constructed: (1) a mean age model; and (2) a female proportion model. Mean age was expressed in years, and female proportion was expressed as a percentage (0–100%).

To standardize the covariates and facilitate subsequent analyses, all reported age data were converted into a study-level mean age. Among the 30 included studies listed in the study characteristics table, age was reported in different formats: most studies provided mean age (typically mean ± standard deviation), some reported only an age range, and two studies reported median age together with minimum–maximum values. When the mean age and sample size were reported separately for the intervention and control groups, the study-level mean age was calculated as a sample size–weighted average across the two groups. If only an overall mean age was reported, that value was used directly. For studies reporting age as a median with minimum–maximum values and with relatively large sample sizes, the recommendations of Hozo et al. (51) were followed, whereby the median was treated as an approximation of the mean, and then combined using sample size weighting where appropriate (51). For studies that reported only a closed age interval without further distributional information, the midpoint of the range was used as an approximation of the mean. For studies with an open-ended range (e.g., studies including only adults), the midpoint could not be directly determined; therefore, additional mean age information was preferentially sought from the original article. If such information was unavailable, the study-level mean age was treated as missing and was not included in the corresponding meta-regression. The resulting study-level mean age estimates were then entered as continuous covariates into the random-effects meta-regression analyses.

#### 2.7.3 Assessment of heterogeneity

Between-study heterogeneity was assessed using Cochran’s *Q* test (with a significance level of *α* = 0.10) and the *I*^2^ statistic. The *I*^2^ value represents the proportion of total variability attributable to true differences in effect sizes rather than sampling error, with 25, 50, and 75% conventionally indicating low, moderate, and high heterogeneity, respectively. When substantial heterogeneity was detected (*p* < 0.10 for Cochran’s *Q* test or *I*^2^ > 50%), potential sources of heterogeneity were further explored through subgroup analyses and meta-regression.

#### 2.7.4 Sensitivity analysis

To examine the robustness of the pooled effect estimates, a leave-one-out sensitivity analysis was conducted. Specifically, each included study was removed sequentially, and the pooled log-transformed relative effect estimate was recalculated to determine whether any single study had a substantial influence on the overall pooled estimate. In addition, a sensitivity analysis was performed after excluding studies with unclear or unadjusted cluster adjustment to evaluate whether the main findings were robust to potential clustering-related precision bias. To clarify the handling of cluster-based studies, each study was categorized according to the availability of knee-specific cluster-adjusted estimates. The cluster-adjustment categories and study-specific adjustment status are provided in Supplementary Appendix 2, Tables S3, S4.

To evaluate whether the findings were sensitive to effect-estimate type, an additional sensitivity analysis was conducted according to the effect estimate actually used in the primary meta-analysis. Event-count-based ORs and reported RRs were classified as risk-based estimates, whereas reported IRRs and rate ratios based on exposure denominators were classified as rate-based estimates. Each category was synthesized separately using a random-effects model, and a mixed-effects subgroup test was used to assess differences between effect-estimate types. Because age moderation was a principal objective of the review, the age subgroup analysis and random-effects meta-regression analyses for study-level mean age and female proportion were additionally repeated after restricting the dataset to risk-based estimates. Moderator analyses were not repeated within the rate-based subset because the limited number and age distribution of the available studies did not permit meaningful age-related analyses. Detailed results are provided in Supplementary Appendix 3, Tables S4, S5.

#### 2.7.5 Publication bias

Publication bias was initially assessed visually using a funnel plot. For pooled analyses including at least 10 studies (*k* ≥ 10), Egger’s regression test and Begg’s rank correlation test were further applied to quantitatively evaluate funnel plot asymmetry and potential small-study effects. The Duval and Tweedie trim-and-fill method was additionally applied to examine whether adjustment for potentially missing studies changed the pooled effect estimate. In addition, the fail-safe N was reported to assess the robustness of the findings. All publication bias analyses were conducted using two-tailed tests.

## 3 Results

### 3.1 Literature search results

**Figure 1:**
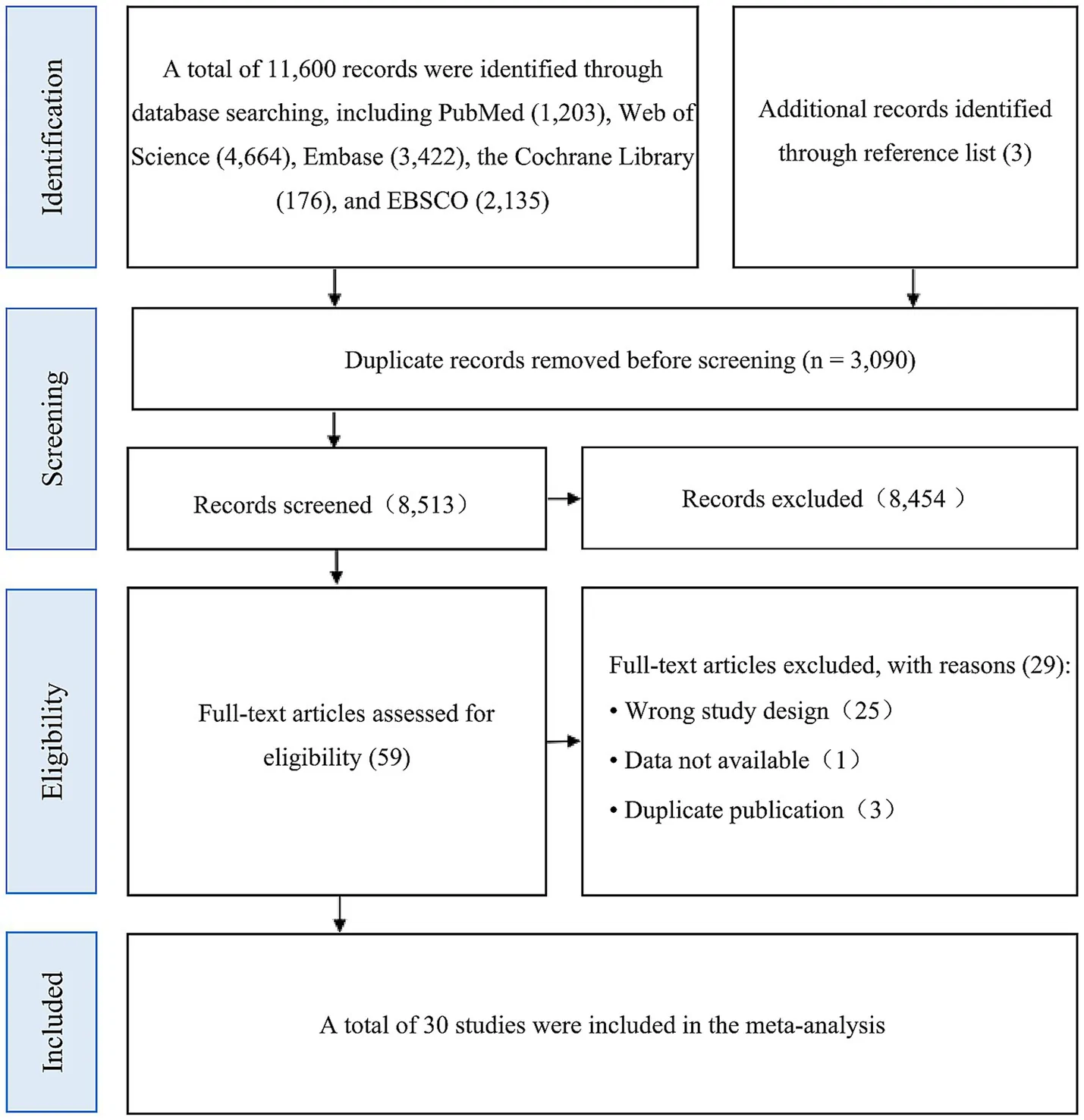
PRISMA flow diagram of the study selection process.

The study selection process is shown in Figure 1. After duplicate removal and eligibility screening, 30 studies were included in the meta-analysis. Inter-reviewer agreement was substantial for title/abstract screening (*κ* = 0.81) and strong for full-text eligibility assessment (κ = 0.88). Inter-reviewer agreement for risk-of-bias assessment was substantial (κ = 0.78), indicating acceptable consistency between the two independent reviewers before consensus discussion.

### 3.2 Characteristics of the included studies

**Figure 2:**
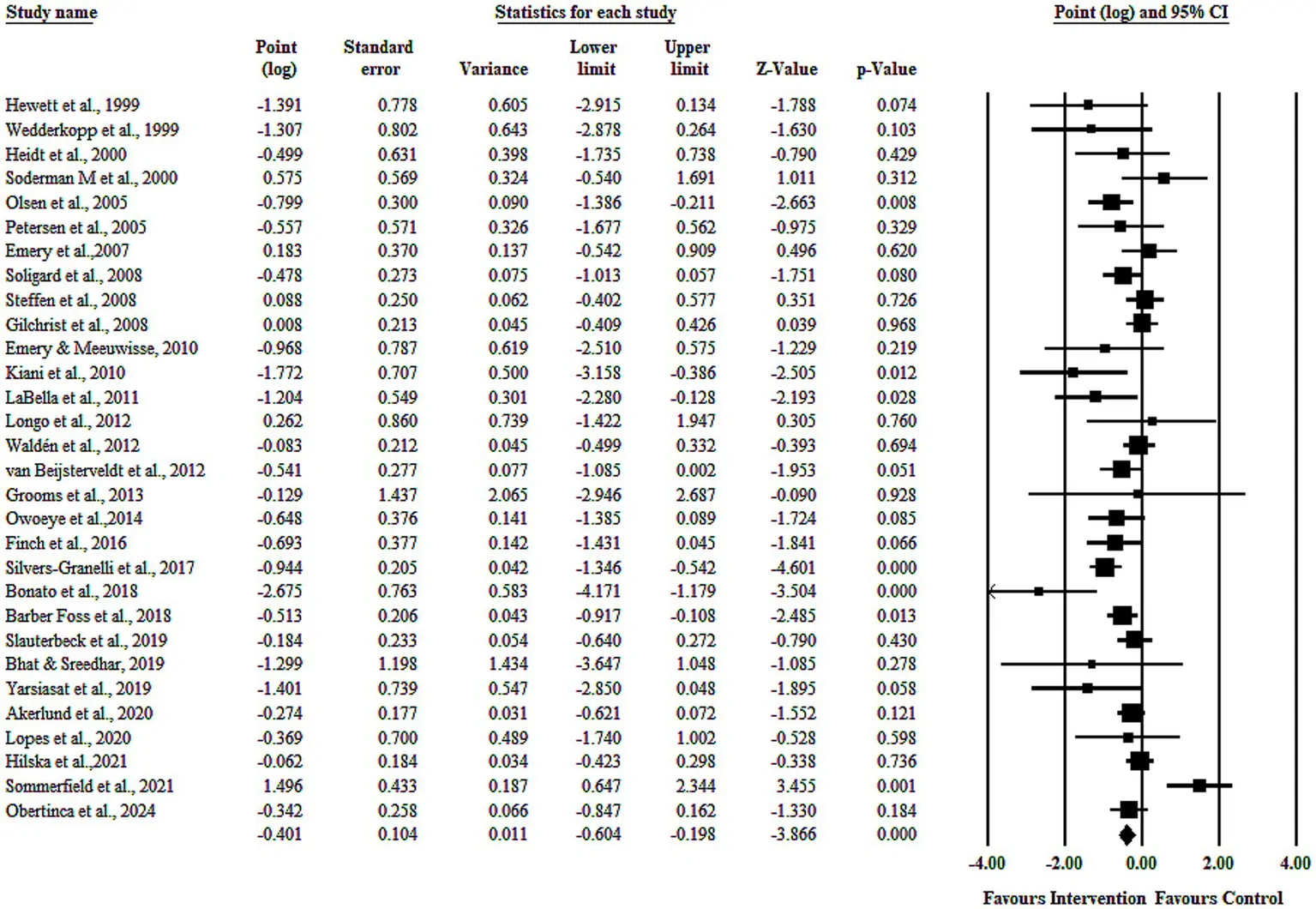
Forest plot of the overall effect of neuromuscular training on the risk of knee injuries. Forest plot of log-transformed effect estimates for the overall effect of neuromuscular training on knee injury risk. Negative values favour the intervention. The pooled log effect was −0.401, 95% CI − 0.604 to −0.198, *p* < 0.001, corresponding to a back-transformed effect estimate of 0.670, 95% CI 0.547–0.820. Heterogeneity: *I*^2^ = 58.5%, *p* < 0.001.

The basic characteristics of the included studies are presented in Table 2, and more detailed intervention-related characteristics and the list of included studies are provided in Supplementary Appendix 2. A total of 30 studies were included, comprising 23 randomized controlled trials and 7 non-randomized controlled trials. Sample sizes varied substantially across studies, and participant age ranged from adolescence to adulthood, although most studies were conducted in adolescent athletes. The included samples comprised female-only, male-only, and mixed-sex populations. Most studies were conducted in team-ball sports, with soccer being the most frequently represented sport, followed by basketball and handball; other sports included volleyball, rugby, sepak takraw, futsal, floorball, and mixed school-based sport samples (Figure 2).

**Table 2:** Basic characteristics of the studies included in the meta-analysis.

Author (Year)	Study design	Evidence level	Participants (EG/CG)	Age (years)	Sport	Knee injuries (EG/CG)
Hewett et al. (21)	NRCT	II	EG:366 CG:463	14–18	Soccer, volleyball, and basketball(W)	EG:2 CG:10
Wedderkopp et al. (73)	RCT	I	EG:111 CG:126	16–18	Handball(W)	EG:2 CG:8
Heidt et al. (84)	NRCT	II	EG:42 CG:258	14–18	Soccer(W)	EG:3 CG:29
Söderman et al. (85)	RCT	I	EG:62 CG:78	EG:20.4 ± 4.6 CG:20.5 ± 5.4	Soccer(W)	EG:8 CG:6
Olsen et al. (70)	RCT	I	EG:958 CG:879	EG:16.3 ± 0.6 CG:16.2 ± 0.6	Handball (F/M)	EG:19 CG:38
Petersen et al. (71)	NRCT	II	EG:134 CG:142	NR (adults)	Handball(W)	EG:5 CG:9
Emery et al. (72)	RCT	I	EG:494 CG:426	EG:16 [13–18] CG:16[12–18]	Basketball (F/M)	EG:18 CG:13
Soligard et al. (34)	RCT	I	EG:1055 CG:837	15.4 ± 0.7	Soccer(W)	EG:33 CG:47
Steffen et al. (32)	RCT	I	EG:1073 CG:947	15.4 ± 0.8	Soccer(W)	EG:37 CG:30
Gilchrist et al. (61)	RCT	I	EG:583 CG:852	19.88	Soccer(W)	EG:40 CG:58
Emery and Meeuwisse (79)	RCT	I	EG:380 CG:364	13–18	Soccer (F/M)	EG:3 CG:8
Kiani et al. (80)	NRCT	II	EG:777 CG:729	EG 14.7 [12.7–18.6] CG 15.0 [13.0–17.6]	Soccer(W)	EG:3 CG:13
LaBella et al. (86)	RCT	I	EG:737 CG:755	EG:16.19 ± 1.53 CG:16.22 ± 1.06	Soccer and basketball(W)	EG:8 CG:17
Longo et al. (62)	RCT	I	EG:80 CG:41	EG:13.5 ± 2.3 CG:15.2 ± 4.6	Basketball(M)	EG:5 CG:2
Waldén et al. (63)	RCT	I	EG:2479 CG:2085	12–17	Soccer(W)	EG:48 CG:44
van Beijsterveldt et al., (64)	RCT	I	EG:223 CG:233	EG: 24.4 ± 4.1 CG: 25.1 ± 4.3	Soccer (M)	EG:24 CG:40
Grooms et al. (83)	NRCT	II	EG:34 CG:30	20.1 ± 2.0	Soccer (M)	EG:1 CG:1
Owoeye et al. (65)	RCT	I	EG:212 CG:204	EG:17.8 ± 0.94 CG:17.5 ± 1.10	Soccer (M)	EG:12 CG:21
Finch et al. (66)	RCT	I	EG:679 CG:885	≥18	Rugby (M)	EG:32 CG:60
Silvers-Granelli et al. (76)	RCT	I	EG:675 CG:850	EG:20 ± 2 CG:21 ± 1	Soccer (M)	EG:34 CG:102
Bonato et al. (74)	RCT	I	EG:86 CG:74	EG:20 ± 2 CG:20 ± 1	Basketball(W)	EG:2 CG:19
Barber Foss et al. (75)	RCT	I	EG:259 CG:215	14.0 ± 1.7	Basketball, soccer, and volleyball(W)	EG:60 CG:72
Slauterbeck et al. (67)	RCT	I	EG:1825 CG:1786	14–18	American football (M), soccer (M/F), basketball (M/F), and hockey (M/F)	EG:35 CG:41
Bhat and Sreedhar (68)	NRCT	II	EG:23 CG:21	EG:22.22 CG:22.05	Basketball (M)	EG:1 CG:3
Yarsiasat et al. (69)	RCT	I	EG:26 CG:26	EG 15.50 ± 1.10 CG: 15.19 ± 1.26	Sepak takraw(W)	EG:3 CG:9
Åkerlund et al. (77)	RCT	I	EG:301 CG:170	EG:13.6 ± 1.1 CG:13.2 ± 1.3	Floorball (F/M)	EG:87 CG:57
Lopes et al. (78)	RCT	I	EG:31 CG:34	EG: 27.7 ± 5.0 CG: 25.8 ± 4.9	Futsal (M)	EG:4 CG:6
Hilska et al. (81)	RCT	I	EG:673 CG:730	9–14	Soccer (F/M)	EG:61 CG:70
Sommerfield et al. (82)	NRCT	II	EG:53 CG:50	EG: 13.9 ± 0.6 CG: 14.1 ± 0.5	Netball, soccer, field hockey, lacrosse, swimming, track and field, badminton, and rowing(W)	EG:31 CG:12
Obërtinca et al. (60)	RCT	I	EG:524 CG:503	EG: 15.2 ± 1.6 CG:15.3 ± 1.6	Soccer (M)	EG:26 CG:36

Age is presented in the order of EG/CG. A single value indicates the mean. a–b indicates the age range. *x* ± *y* indicates the mean ± standard deviation. x [a–b] indicates the median [a–b]. RCT = randomized controlled trial; NRCT = non-randomized controlled trial. F = female; M = male.

### 3.3 Risk-of-bias assessment

The results of the risk-of-bias assessment are presented in Tables 3, 4. Among the 23 randomized controlled trials assessed using RoB 2, no study was judged as having low overall risk of bias, 19 studies were judged as having some concerns, and 4 studies were judged as having high risk of bias. The main sources of concern were incomplete reporting of the randomization process, potential deviations from intended interventions, outcome measurement issues, and selective reporting concerns. Additional concerns related to missing outcome data were observed in some studies.

**Table 3:** Risk-of-bias assessment of randomized controlled trials using the Cochrane RoB 2 tool.

Study	1	2	3	4	5	Overall
Wedderkopp et al. (73)	SC	SC	L	SC	SC	SC
Söderman et al. (85)	SC	H	H	SC	SC	H
Olsen et al. (70)	SC	SC	L	L	SC	SC
Emery et al. (72)	SC	SC	SC	L	SC	SC
Soligard et al. (34)	SC	SC	SC	L	L	SC
Steffen et al. (32)	L	SC	L	L	SC	SC
Gilchrist et al. (61)	SC	H	H	SC	SC	H
Emery and Meeuwisse (79)	SC	SC	SC	L	SC	SC
LaBella et al. (86)	L	SC	L	SC	L	SC
Longo et al. (62)	SC	SC	L	SC	SC	SC
Waldén et al. (63)	L	SC	SC	L	L	SC
van Beijsterveldt et al. (64)	SC	SC	L	SC	L	SC
Owoeye et al. (65)	SC	SC	L	SC	SC	SC
Finch et al. (66)	SC	SC	SC	L	L	SC
Silvers-Granelli et al. (76)	SC	H	H	SC	SC	H
Bonato et al. (74)	SC	SC	L	SC	SC	SC
Barber Foss et al. (75)	SC	SC	L	SC	SC	SC
Slauterbeck et al. (67)	SC	SC	L	SC	SC	SC
Yarsiasat et al. (69)	SC	SC	L	SC	SC	SC
Lopes et al. (78)	SC	SC	SC	SC	SC	SC
Hilska et al. (81)	L	SC	SC	SC	L	SC
Åkerlund et al. (77)	SC	SC	H	SC	L	H
Obërtinca et al. (60)	L	SC	SC	SC	L	SC

RoB 2, revised Cochrane Risk of Bias tool for randomized trials. In the present study, randomized controlled trials were assessed using five domains of RoB 2. In the table, items 1–5 represent the five risk-of-bias domains of RoB 2: 1, bias arising from the randomization process; 2, bias due to deviations from intended interventions; 3, bias due to missing outcome data; 4, bias in measurement of the outcome; 5, bias in selection of the reported result. For cluster-randomized trials, the additional RoB 2 domain concerning the timing of identification and recruitment of participants within clusters was considered where applicable. Each domain and the overall judgment were rated as low risk of bias, some concerns, or high risk of bias. The abbreviations used in the table are as follows: L, low risk of bias; SC, some concerns; H, high risk of bias.

**Table 4:** Risk-of-bias assessment of non-randomized controlled intervention studies using ROBINS-I.

Study	1	2	3	4	5	6	7	Overall
Hewett et al. (21)	S	M	L	M	L	M	NI	S
Heidt et al. (84)	S	M	L	M	NI	L	NI	S
Kiani et al. (80)	M	M	L	M	L	L	NI	M
Grooms et al. (83)	S	M	L	L	L	M	NI	S
Petersen et al. (71)	S	M	L	M	M	M	NI	S
Bhat and Sreedhar (68)	S	S	M	M	M	S	NI	S
Sommerfield et al. (82)	S	S	L	M	M	S	S	S

ROBINS-I, Risk Of Bias In Non-randomized Studies of Interventions. Non-randomized controlled intervention studies were assessed using the original 2016 ROBINS-I tool across seven domains: 1, bias due to confounding; 2, bias in selection of participants into the study; 3, bias in classification of interventions; 4, bias due to deviations from intended interventions; 5, bias due to missing data; 6, bias in measurement of outcomes; 7, bias in selection of the reported result. Each domain and the overall judgment were rated as low, moderate, serious, critical, or no information. The overall judgment was not calculated as a total score, but was determined according to the ROBINS-I guidance, taking into account the most serious domain-level judgment. L, low risk; M, moderate risk; S, serious risk; C, critical risk; NI, no information.

Among the 7 non-randomized controlled intervention studies assessed using ROBINS-I, one study was judged as having moderate overall risk of bias and six studies were judged as having serious overall risk of bias. Inter-reviewer agreement for risk-of-bias assessment was substantial (*κ* = 0.78), indicating acceptable consistency between the two independent reviewers before consensus discussion.

### 3.4 Meta-analysis results

Complete sensitivity and publication-bias analyses are provided in Supplementary Appendix 3.

#### 3.4.1 Overall effect of neuromuscular training on knee injury risk

The primary random-effects meta-analysis included 30 studies. Neuromuscular training was associated with a significant reduction in knee injury risk among athletes. The pooled log-transformed effect estimate was −0.401 (95% CI: −0.604 to −0.198; *p* < 0.001), corresponding to a back-transformed effect estimate of 0.670 (95% CI: 0.547 to 0.820). The direction of effect favoured the intervention group, indicating that neuromuscular training was associated with a lower risk of knee injury compared with the control condition. Moderate heterogeneity was observed (*I*^2^ = 58.5%; *p* < 0.001).

#### 3.4.2 Subgroup analyses

##### 3.4.2.1 Age

As shown in Table 5, neuromuscular training was associated with a significant reduction in knee injury risk in both adult athletes (>18 years: *k* = 10; effect estimate = 0.566, 95% CI: 0.367 to 0.873; *p* = 0.010) and adolescent athletes (≤18 years: *k* = 20; effect estimate = 0.718, 95% CI: 0.572 to 0.902; *p* = 0.004). The between-subgroup difference was not statistically significant (*p* = 0.340), indicating that no clear study-level age-category moderation was detected.

**Table 5:** Age subgroup analyses of neuromuscular training for preventing knee injuries.

Analysis	Subgroup	*k*	Log effect	95% CI	Back-transformed effect estimate (95% CI)	*p*-value	*p* for subgroup difference	*I*^2^ (%)	*p* for heterogeneity
All athletes	>18 years	10	−0.569	−1.002 to −0.136	0.566 (0.367–0.873)	0.010	0.340	60.6	0.007
	≤18 years	20	−0.331	−0.558 to −0.103	0.718 (0.572–0.902)	0.004	—	56.2	0.001
Male athletes	>18 years	6	−0.767	−1.053 to −0.481	0.464 (0.349–0.618)	<0.001	0.146	0.0	0.826
	≤18 years	3	−0.400	−0.804 to 0.005	0.670 (0.448–1.005)	0.053	—	0.0	0.583
Female athletes	>18 years	3	−0.563	−2.004 to 0.877	0.569 (0.135–2.404)	0.443	0.879	84.7	0.001
	≤18 years	11	−0.446	−0.886 to −0.007	0.640 (0.412–0.993)	0.047	—	71.5	<0.001

Back-transformed effect estimates were calculated by exponentiating the log effect estimates and their corresponding 95% confidence intervals. Values below 1.00 indicate a lower risk of knee injury in the neuromuscular training group.

In sex-stratified age subgroup analyses, the preventive effect was significant in adult males but did not reach statistical significance in adolescent males, with no significant difference between age subgroups (*p* = 0.146). Among female athletes, the adult subgroup showed a non-significant effect with a wide confidence interval, whereas the adolescent subgroup showed a significant reduction in knee injury risk; however, the between-subgroup difference was not statistically significant (*p* = 0.879).

##### 3.4.2.2 Training components

In the subgroup analysis of training components (Table 6), interventions incorporating landing-stabilization training, plyometric training, core training, agility training, or lower-limb strength training showed significant preventive effects, with pooled effect estimates ranging from 0.599 to 0.664. In contrast, the corresponding subgroups without these components did not reach statistical significance. For stretching and balance training, significant preventive effects were observed in both the present and absent subgroups. However, none of the between-subgroup differences for individual training components reached statistical significance (*p* = 0.104–0.790), suggesting that these component-specific findings should be interpreted as exploratory rather than as evidence of clear effect modification.

**Table 6:** Subgroup analyses by training components and program characteristics of neuromuscular training for preventing knee injuries.

Analysis	Subgroup	*k*	Log effect	95% CI	Back-transformed effect estimate (95% CI)	*p*-value	*p* for subgroup difference	*I*^2^ (%)	*p* for heterogeneity
Landing stabilization	Absent	6	−0.322	−0.879 to 0.235	0.725 (0.415–1.265)	0.257	0.790	41.4	0.129
	Present	15	−0.409	−0.725 to −0.092	0.664 (0.484–0.912)	0.011	—	70.5	<0.001
Plyometric training	Absent	5	−0.106	−0.584 to 0.372	0.899 (0.558–1.451)	0.664	0.205	25.8	0.249
	Present	25	−0.447	−0.670 to −0.224	0.640 (0.512–0.799)	<0.001	—	61.6	<0.001
Core training	Absent	8	−0.208	−0.617 to 0.201	0.812 (0.540–1.223)	0.320	0.333	24.8	0.232
	Present	22	−0.440	−0.671 to −0.208	0.644 (0.511–0.812)	<0.001	—	63.9	<0.001
Agility training	Absent	7	−0.220	−0.442 to 0.002	0.803 (0.643–1.002)	0.053	0.104	20.1	0.276
	Present	23	−0.513	−0.788 to −0.238	0.599 (0.455–0.788)	<0.001	—	63.5	<0.001
Lower-limb strength training	Absent	3	−0.005	−0.872 to 0.861	0.995 (0.418–2.366)	0.991	0.346	47.7	0.148
	Present	26	−0.434	−0.648 to −0.220	0.648 (0.523–0.803)	<0.001	—	60.7	<0.001
Stretching training	Absent	15	−0.274	−0.528 to −0.020	0.760 (0.590–0.980)	0.034	0.139	60.3	0.001
	Present	15	−0.592	−0.928 to −0.256	0.553 (0.395–0.774)	0.001	—	55.1	0.005
Balance training	Absent	3	−0.937	−1.810 to −0.064	0.392 (0.164–0.938)	0.035	0.222	0.0	0.616
	Present	27	−0.377	−0.586 to −0.168	0.686 (0.557–0.845)	<0.001	—	61.2	<0.001
Number of components	<3 components	3	0.199	−0.391 to 0.789	1.220 (0.676–2.201)	0.508	0.045	0.2	0.367
	≥3 components	27	−0.441	−0.650 to −0.232	0.643 (0.522–0.793)	<0.001	—	59.7	<0.001
Training standardization	Standardized programs	19	−0.525	−0.745 to −0.305	0.592 (0.475–0.737)	<0.001	0.066	50.1	0.007
	Non-standardized programs	11	−0.120	−0.491 to 0.252	0.887 (0.612–1.287)	0.527	0.066	55.6	0.013

Back-transformed effect estimates were obtained by exponentiating the log effect estimates and their corresponding 95% confidence intervals. Values below 1.00 indicate a lower risk of knee injury in the neuromuscular training group.

##### 3.4.2.3 Number of training components

For the number of training components (Table 6), interventions including three or more components showed a significant reduction in knee injury risk (effect estimate = 0.643, 95% CI 0.522–0.793, *p* < 0.001), whereas interventions with fewer than three components did not show a significant effect (effect estimate = 1.220, 95% CI 0.676–2.201, *p* = 0.508). The between-subgroup difference was statistically significant (*p* = 0.045), suggesting that multicomponent neuromuscular training programs may be associated with greater preventive effects. However, this finding should be interpreted cautiously because the subgroup with fewer than three components included only three studies.

##### 3.4.2.4 Training standardization

The subgroup analysis according to training standardization (Table 6) showed that standardized neuromuscular training programs significantly reduced the risk of knee injuries (*k* = 19; log effect = −0.525, 95% CI: −0.745 to −0.305; back-transformed effect estimate = 0.592, 95% CI: 0.475–0.737; *p* < 0.001; *I*^2^ = 50.1%). In contrast, non-standardized programs did not show a statistically significant preventive effect (*k* = 11; log effect = −0.120, 95% CI: −0.491 to 0.252; back-transformed effect estimate = 0.887, 95% CI: 0.612–1.287; *p* = 0.527; *I*^2^ = 55.6%). The between-subgroup difference approached but did not reach statistical significance (p for subgroup difference = 0.066), suggesting that standardized programs may be associated with a larger and more consistent protective effect, although this finding should be interpreted cautiously as exploratory evidence.

##### 3.4.2.5 Training dose

In the subgroup analyses of training dose (Table 7), no significant between-subgroup differences were observed for session duration, weekly frequency, or intervention duration, suggesting no clear evidence that these dose-related variables modified the preventive effect of neuromuscular training. For session duration, only the 15–20 min/session subgroup showed a significant preventive effect and included the largest number of studies (effect estimate = 0.642, 95% CI: 0.527–0.783, *p* < 0.001), whereas the <15 min/session and >20 min/session subgroups did not reach statistical significance. For weekly frequency, both <2 sessions/week and 2–3 sessions/week significantly reduced knee injury risk, whereas >3 sessions/week were not statistically significant. The 2–3 sessions/week subgroup showed a stable preventive estimate (effect estimate = 0.666, 95% CI: 0.531–0.835, *p* < 0.001). For intervention duration, both <12 weeks and >25 weeks showed significant risk reductions, whereas 12–25 weeks did not. Although the <12-week subgroup showed the lowest pooled effect estimate, it included only four studies and should therefore be interpreted cautiously. Interventions lasting >25 weeks also showed a significant preventive effect based on a larger number of studies (effect estimate = 0.643, 95% CI: 0.488–0.848, *p* = 0.002). Overall, although none of the dose variables showed a statistically significant subgroup effect, the results suggested that 15–20 min/session, 2–3 sessions/week, and intervention duration longer than 25 weeks represented relatively stable and clinically interpretable dose patterns (Table 8).

**Table 7:** Training-dose subgroup analyses of neuromuscular training for preventing knee injuries.

Dose variable	Subgroup	*k*	Log effect	95% CI	Back-transformed effect estimate (95% CI)	*p*-value	*p* for subgroup difference	*I*^2^ (%)	*p* for heterogeneity
Session duration	<15 min/session	6	−0.340	−0.711 to 0.030	0.712 (0.491–1.030)	0.072	0.880	31.7	0.198
	15–20 min/session	16	−0.443	−0.640 to −0.245	0.642 (0.527–0.783)	<0.001	—	33.2	0.096
	>20 min/session	6	−0.489	−1.282 to 0.304	0.613 (0.277–1.355)	0.227	—	85.1	<0.001
Weekly frequency	<2 sessions/week	9	−0.406	−0.702 to −0.109	0.666 (0.496–0.897)	0.007	0.877	46.7	0.059
	2–3 sessions/week	11	−0.407	−0.633 to −0.180	0.666 (0.531–0.835)	<0.001	—	40.9	0.076
	>3 sessions/week	7	−0.600	−1.319 to 0.119	0.549 (0.267–1.126)	0.102	—	64.9	0.009
Intervention duration	<12 weeks	4	−1.049	−1.806 to −0.291	0.350 (0.164–0.748)	0.007	0.160	0.0	0.747
	12–25 weeks	11	−0.219	−0.612 to 0.174	0.803 (0.542–1.190)	0.275	—	72.7	<0.001
	>25 weeks	13	−0.441	−0.718 to −0.165	0.643 (0.488–0.848)	0.002	—	54.7	0.009

Back-transformed effect estimates were obtained by exponentiating the log effect estimates and their corresponding 95% confidence intervals. Values below 1.00 indicate a lower risk of knee injury in the neuromuscular training group.

**Table 8:** Random-effects meta-regression analyses of age and female proportion as moderators of neuromuscular training effects on knee injury prevention.

Moderator	*k*	Coefficient *β*	SE	95% CI	*Z*-value	*p*-value
Mean age	28	−0.042	0.032	−0.105 to 0.020	−1.33	0.185
Female proportion (%)	28	0.001	0.003	−0.004 to 0.006	0.23	0.821

k, number of studies included in the meta-regression; SE, standard error; CI, confidence interval. The coefficient *β* represents the change in the log-transformed effect estimate associated with a one-unit increase in the moderator.

#### 3.4.3 Meta-regression analyses

Random-effects meta-regression analyses were performed to examine whether study-level mean age and female proportion moderated the effect of neuromuscular training on knee injury prevention. Meta-regression did not detect clear evidence that study-level mean age moderated the intervention effect (*β* = −0.042; SE = 0.032; 95% CI: −0.105 to 0.020; *p* = 0.185). Similarly, female proportion was not significantly associated with the intervention effect (*β* = 0.001; SE = 0.003; 95% CI: −0.004 to 0.006; *p* = 0.821). These findings suggest that study-level mean age and sex composition were not significant moderators of the pooled effect estimates.

#### 3.4.4 Sensitivity analysis

Sensitivity analyses generally supported the robustness of the pooled findings. In the leave-one-out analysis, the back-transformed pooled estimates ranged from 0.645 to 0.697 and remained statistically significant after each study was sequentially excluded (all *p* < 0.001). After excluding studies with unclear or unadjusted cluster adjustment, the overall pooled estimate also remained statistically significant (*k* = 14; effect estimate = 0.611, 95% CI: 0.438–0.852; *p* = 0.004; *I*^2^ = 60.7%). The age-subgroup difference remained non-significant (*p* = 0.473), and neither mean age (*β* = −0.211, *p* = 0.082) nor female proportion (*β* = 0.001, *p* = 0.835) significantly moderated the intervention effect. In the sensitivity analysis stratified by effect-estimate type, significant protective effects were observed for both risk-based ORs/RRs (*k* = 24; effect estimate = 0.687, 95% CI: 0.532–0.890; p = 0.004) and exposure-based IRRs/rate ratios (*k* = 6; effect estimate = 0.654, 95% CI: 0.519–0.823; *p* < 0.001). The difference between the two categories was not statistically significant (Q_between = 0.082, df = 1, *p* = 0.775; Supplementary Appendix 3, Table S4).

When restricted to risk-based estimates, the protective effect was significant in athletes aged >18 years (*k* = 9; effect estimate = 0.572, 95% CI: 0.349–0.939; *p* = 0.027) and directionally protective but non-significant in athletes aged ≤18 years (*k* = 15; effect estimate = 0.751, 95% CI: 0.556–1.016; *p* = 0.064). However, the between-age-group difference remained non-significant (*p* = 0.357). Neither mean age (*β* = −0.047, *p* = 0.204) nor female proportion (*β* = 0.001, *p* = 0.688) was a significant moderator in the risk-based subset (Supplementary Appendix 3, Table S5). Overall, these analyses supported the robustness of the protective direction of NMT and the conclusion that no clear study-level age moderation was detected.

#### 3.4.5 Publication bias analysis

Publication bias was assessed for the overall pooled effect using Egger’s regression test, Begg and Mazumdar rank correlation test, Duval and Tweedie trim-and-fill analysis, and the classic fail-safe N method. Egger’s regression test did not indicate significant small-study effects (intercept = −0.877, 95% CI: −2.054 to 0.299, *p* = 0.138). Similarly, Begg and Mazumdar rank correlation test showed no significant evidence of publication bias, either without continuity correction (Kendall’s tau = −0.186, *p* = 0.148) or with continuity correction (Kendall’s tau = −0.184, *p* = 0.154). The trim-and-fill analysis imputed no missing studies, and the adjusted random-effects log estimate remained unchanged from the observed estimate (−0.401, 95% CI: −0.604 to −0.198). In addition, the classic fail-safe N indicated that 271 missing studies would be required to make the pooled effect non-significant. Overall, these findings suggest no clear evidence of substantial publication bias in the overall analysis.

## 4 Discussion

This systematic review and meta-analysis synthesized evidence from 30 prospective studies to comprehensively evaluate the overall effectiveness of neuromuscular training (NMT) in preventing sports-related knee injuries, and to further examine the potential moderating roles of age and training-program characteristics. The overall findings showed that NMT significantly reduced the risk of knee injuries in athletes. Further analyses of potential moderators did not detect clear study-level age moderation of the preventive effect of NMT. Exploratory analyses suggested that training-program characteristics, including the number and combination of training components, protocol standardization, and dose-related patterns, may be associated with more favorable or more stable preventive estimates. Based on these findings, the following discussion is organized around two main dimensions, namely age-related findings and training-related implementation characteristics, with further interpretation of the possible mechanisms and practical implications.

### 4.1 Comparison with previous research and novel contribution

Compared with previous systematic reviews and meta-analyses, which have mainly focused on ACL injuries, female or youth athletes, lower-extremity injuries, or exercise-based injury prevention in general (35, 36, 39, 40), the present study provides a more knee-specific and implementation-oriented perspective. By examining age category, study-level mean age, sex composition, training components, number of components, program standardization, and dose-related characteristics within the same analytical framework, this review extends previous work beyond the question of whether NMT is effective. The novel contribution of this study is to frame NMT not only as an effective injury-prevention strategy, but also as an intervention whose practical success may depend on how it is structured, delivered, and sustained in real-world sport settings.

### 4.2 Age-related findings and study-level moderators

The present study showed that both adolescent athletes (≤18 years) and adult athletes (>18 years) experienced significant reductions in knee injury risk following neuromuscular training (NMT), with no statistically significant difference between age subgroups. Meta-regression analysis likewise did not detect clear evidence that study-level mean age moderated the intervention effect. This finding should be interpreted in the context of previous ACL-specific literature, in which theoretical and empirical work has suggested that adolescents, particularly female athletes, may derive greater benefit from neuromuscular injury-prevention programs because of age- and maturation-related differences in neuromuscular plasticity (40, 41, 52). However, the outcome of the present study was overall knee injury, and the included injury types may have encompassed ACL injuries, meniscal injuries, medial and lateral collateral ligament injuries, and patellofemoral joint-related injuries. Different injury types may vary in both their underlying mechanisms and their dependence on improvements in neuromuscular control. Consequently, age-related signals observed at the ACL-specific level may have been diluted when evaluated within the broader and more heterogeneous outcome of overall knee injuries, resulting in the absence of a stable age-related moderating effect at the pooled level. At the same time, even under relatively homogeneous outcomes, the key mechanisms through which NMT exerts its effects—such as movement pattern learning, dynamic postural control, and improvements in neuromuscular control—can be achieved across different age groups. Age-related differences may be more likely to manifest in the rate of adaptation or the training dose required, rather than in statistically detectable differences in pooled effect sizes within the range of populations and protocols currently represented in the available evidence. Overall, NMT can therefore be regarded, at the present stage, as a preventive strategy applicable across age groups, providing an evidence-based basis for the implementation of structured injury prevention programs in athletes of different ages.

Meta-regression further examined whether study-level mean age and the proportion of female participants were associated with the preventive effect of NMT. Neither variable showed a significant moderating effect, suggesting that study-level mean age and female proportion did not clearly modify the preventive effect of NMT. These findings were consistent with the age subgroup analyses and indicate that, within the range of populations included in the current evidence, age and sample sex composition did not show clear study-level associations with intervention effectiveness.

### 4.3 Outcome definitions, effect measures, surveillance, and heterogeneity

An additional methodological issue that should be considered when interpreting the pooled findings is the variation in injury definitions and injury-surveillance procedures across the included studies. Although all studies reported knee-related injury outcomes, the operational definitions of injury differed substantially. Some studies adopted time-loss definitions, whereas others included medically attended injuries, clinically diagnosed injuries, self-reported physical complaints, or broader injury events recorded during training and competition. Consequently, studies using more restrictive definitions may have captured primarily moderate-to-severe injuries, whereas studies using broader surveillance systems may also have included minor or transient knee problems. In addition, surveillance procedures varied across studies and included physicians, physiotherapists, athletic trainers, coaches, researchers, injury-reporting systems, and athlete self-report methods. Differences in surveillance source and, where reported, diagnostic confirmation may have influenced injury ascertainment, reporting accuracy, and classification consistency. Furthermore, recurrent injuries were handled differently across studies, with some studies including recurrent injuries and others focusing only on first-time injuries. These methodological differences may have contributed to between-study heterogeneity and should be considered when interpreting the pooled estimates. Therefore, the pooled effect should be interpreted as reflecting the overall preventive effect of neuromuscular training on reported knee-injury outcomes rather than on a single standardized injury definition. To improve transparency, injury definitions, surveillance characteristics, exposure information, recurrent-injury handling, and injury-counting approaches for all included studies are summarized in Supplementary Appendix 2, Table S2.

The use of different relative effect measures should also be considered when interpreting the pooled estimate. ORs and RRs generally reflect injured versus non-injured participants or cumulative injury risk, whereas IRRs and rate ratios use exposure-time denominators and may reflect total or recurrent injury events. Therefore, although these ratio measures can be analysed on the natural logarithmic scale using a generic inverse-variance approach, they are not statistically identical and do not necessarily answer exactly the same clinical question. For this reason, the primary pooled estimate was interpreted as a summary relative effect, without assigning it to a single effect-measure category. In the effect-estimate-type sensitivity analysis, risk-based ORs/RRs and exposure-based IRRs/rate ratios both showed significant protective effects, with no significant difference between the two categories. This finding does not establish equivalence between the measures, but indicates that the overall protective conclusion was not materially altered when the two effect-estimate types were analysed separately. Moreover, after restricting the age analyses to risk-based estimates, neither the between-age-group difference nor the continuous mean-age meta-regression was statistically significant, indicating that the conclusion regarding the absence of clear study-level age moderation was not dependent on the inclusion of rate-based estimates.

### 4.4 Program composition, standardization, and implementation quality

In contrast to age, the present findings suggest that the composition of training programs may be associated with differences in intervention effectiveness. Subgroup analyses showed that multicomponent NMT programs including at least three training components produced significant preventive effects, whereas programs with fewer components did not demonstrate significant pooled effects; moreover, the between-group difference reached statistical significance (*p* = 0.045). From a mechanistic perspective, knee injuries are often the result of the interaction of multiple risk factors (14, 53, 54). Therefore, multicomponent programs that combine landing stabilization, strength, core, plyometric, and agility-related elements may reduce injury risk more consistently by targeting movement control and neuromuscular function through multiple pathways (45, 53). By contrast, programs with relatively limited content may improve only selected capacities and may therefore have restricted ability to address the complex combination of risks encountered in dynamic sporting situations, resulting in reduced preventive benefit (23). It should be emphasized that a multicomponent approach does not imply simply adding unlimited training volume. Rather, the critical issue is to achieve a rational combination and progression of modules based on injury mechanisms, so as to broaden program coverage while maintaining training quality and feasibility, and at the same time to improve adherence through greater training variety (55).

The present study also found that standardized neuromuscular training programs showed a larger and statistically significant protective effect, whereas non-standardized programs showed a smaller and non-significant effect estimate. Although the between-subgroup difference approached but did not reach statistical significance, the magnitude of the effect estimate and the narrower confidence interval in the standardized subgroup suggest that programs with a clear structure, specified dose, explicit progression, executable technical requirements, and good reproducibility may be more likely to produce stable preventive benefits. Standardized programs typically include clearly defined training modules, progression schedules, and quality-control criteria, thereby reducing variability during implementation and improving consistency of delivery. By contrast, non-standardized programs tend to vary considerably in terms of training content and execution quality, which may contribute to fluctuations in effectiveness and attenuate statistical significance in pooled analyses. Previous studies have similarly suggested that well-structured and validated multicomponent prevention programs can provide stable injury prevention benefits across different populations. For example, programs built around standardized warm-up and preventive training routines have been repeatedly validated in sports such as football and have been associated with lower rates of lower-extremity injuries (39, 42, 56). It should be noted that the greater stability observed in standardized programs may not be attributable solely to the training content itself, but also to better organizational management and implementation quality. When implementation is supported by coach education, supervision, and feedback mechanisms, training adherence is often improved, thereby enhancing intervention effectiveness. Conversely, even when a program has theoretical advantages, its preventive benefit may remain limited if ongoing adherence and quality control are lacking. In the present study, adherence could not be uniformly quantified or formally tested as a moderator because reporting of adherence was inconsistent and incomplete across the included studies. Nevertheless, previous literature has consistently indicated that higher adherence is associated with greater reductions in injury risk (42). Therefore, in practical implementation, it is important not only to ensure the scientific soundness of training structure, but also to emphasize the quality of delivery and continuity of participation, through appropriate scheduling, optimization of program design, and the establishment of supervision and feedback systems, in order to maximize the preventive potential of neuromuscular training.

### 4.5 Training dose and sustained implementation

The dose characteristics of neuromuscular training (NMT) interventions—including session duration, training frequency, and intervention length—may be associated with preventive effectiveness. In the present study, dose-related subgroup analyses indicated that programs lasting approximately 15–20 min per session and performed 2–3 times per week achieved significant reductions in injury risk. Short and moderate session durations are also more feasible for integration into routine training schedules, for example as part of a warm-up, as they can provide sufficient training stimulus while reducing fatigue accumulation associated with longer sessions, thereby helping to maintain movement quality and consistency of execution. At the same time, a training frequency of 2–3 sessions per week is generally considered sufficient to induce beneficial neuromuscular adaptations without imposing excessive time or physical burdens on athletes.

Regarding intervention duration and the seasonal training cycle, although the between-subgroup differences across intervention-length categories did not reach statistical significance in the present study, a longer duration of continuous training (>25 weeks) may be more beneficial for consolidating movement patterns and neuromuscular control, thereby reinforcing training adaptations and maintaining their effects throughout the season. By contrast, although short-term interventions may show relatively large reductions in injury risk in some studies, such findings may be more susceptible to the influence of sample size, implementation intensity, and follow-up duration; therefore, caution is warranted when extrapolating these results to long-term sporting contexts. It has been suggested that the risk of certain high-risk knee injuries may persist throughout the entire season, and that preventive training should therefore emphasize continuous implementation in order to avoid attenuation of effects over time (40, 57). At the same time, extending program duration may be accompanied by declining motivation or fluctuations in execution quality. For this reason, long-term programs may benefit from strategies such as variation in training content, phased feedback, and team-based support to enhance engagement and adherence, thereby ensuring the realization of cumulative benefits. On this basis, we recommend that NMT be incorporated as a regular component of team training on a long-term basis, and that it continue to be reinforced during the off-season or transition periods in order to consolidate the protective effects already achieved (58).

### 4.6 Risk of bias, publication bias, and certainty of interpretation

According to AMSTAR 2, risk of bias and publication bias should be considered when interpreting the findings of systematic reviews and meta-analyses (59). In the present review, most randomized controlled trials were judged as having some concerns, and several were judged as having high risk of bias. In addition, most non-randomized controlled intervention studies were judged as having serious risk of bias. Therefore, although the overall direction of the pooled effect supports the preventive effect of NMT on sports-related knee injuries, the magnitude and certainty of the pooled estimate should be interpreted cautiously. The publication-bias analyses did not show clear evidence of small-study effects or substantial publication bias. However, negative publication-bias tests cannot completely exclude publication bias, especially given the heterogeneity in study design, injury definitions, surveillance procedures, and effect-estimate reporting. Therefore, publication bias does not appear to be a major explanation for the pooled result, but its potential influence cannot be fully ruled out.

## 5 Practical applications

The findings of this review support the practical use of neuromuscular training as a feasible strategy for reducing sports-related knee injury risk in both adolescent and adult athletes. In applied settings, NMT may be implemented as a structured and multicomponent routine rather than as isolated exercises alone. When feasible, programs may incorporate commonly used components such as landing-stabilization training, plyometric training, core training, agility/change-of-direction drills, and lower-limb strength training. Coaches and practitioners should also emphasize standardized delivery, progressive difficulty, technical execution quality, supervision, and program adherence, as implementation quality may influence real-world effectiveness. Based on the available evidence, sessions lasting approximately 15–20 min, performed 2–3 times per week, and sustained for >25 weeks or across a substantial part of the season when feasible may represent a practical reference range. However, because these training characteristics were examined in exploratory analyses, the proposed recommendations should be interpreted as feasible guidance for practice rather than fixed prescriptions.

## 6 Limitations and future directions

Several limitations should be acknowledged. First, the moderator analyses were based on study-level aggregated data rather than individual participant data, which limited the ability to examine individual-level age effects, nonlinear age-response relationships, and potential interactions among age, sex, biological maturation, adherence, and intervention response. Second, injury definitions, surveillance procedures, diagnostic confirmation, and recurrent-injury handling varied across studies, which may have affected outcome comparability and contributed to between-study heterogeneity. Third, the primary synthesis included related but non-identical risk-based and rate-based effect estimates. Although the effect-estimate-specific sensitivity analyses produced consistent protective estimates and did not identify a statistically significant between-category difference, the rate-based subset included only six studies. Moreover, the absence of a statistically significant subgroup difference does not establish equivalence between these estimands. Therefore, the primary pooled estimate should be interpreted as a broad relative effect estimate and considered together with the separate risk-based and rate-based sensitivity analyses. Fourth, some intervention characteristics required classification or data harmonization. Training components were coded according to the descriptions provided in the original studies, which may have been influenced by differences in reporting quality. In addition, dose-related variables required midpoint estimation for dose ranges and weighted averaging across intervention phases. Therefore, findings related to specific training components and dose characteristics should be interpreted as exploratory. Fifth, the classification of standardized versus non-standardized NMT programs was based on prespecified but non-validated criteria, which may have introduced some subjectivity.

Future studies should adopt standardized injury definitions and surveillance procedures, clearly distinguish first-time and recurrent injuries, and report exposure-adjusted outcomes whenever possible. More detailed reporting of training components, progression, supervision, adherence, and implementation fidelity is also needed. Individual participant data meta-analyses and well-designed prospective trials are warranted to clarify whether age, sex, biological maturation, sport type, adherence, and training characteristics modify the effectiveness of NMT.

## 7 Conclusion

This systematic review and meta-analysis showed that neuromuscular training (NMT) significantly reduces the risk of knee injuries in athletes. This protective effect was observed in both adolescent and adult athletes, and no clear study-level age moderation was detected. The findings support the implementation of NMT as a knee injury prevention strategy across athletes of different age groups. Structured, multicomponent, and standardized programs may have practical implementation advantages; however, findings related to program structure, specific training components, standardization, and dose characteristics should be interpreted cautiously because these analyses were exploratory.

## Data Availability

The original contributions presented in the study are included in the article/Supplementary material, further inquiries can be directed to the corresponding author.
