# Supplementary material for: Age-moderating effects and training characteristics of neuromuscular training for preventing knee injuries: a systematic review and meta-analysis
Source: Front Public Health. 2026 Aug 28;14:1822715. doi: 10.3389/fpubh.2026.1822715 (PMC13566741; doi:10.3389/fpubh.2026.1822715)
Supplement: Supplementary file 2 [file Supplementary_file_2.docx]

**Supplementary Appendix 2.**

**Table 1 Characteristics of the included studies**

| **Author/Year** | **Study design** | **Level of evidence** | ****Sample size**(EG/CG)** | **Age (years)** | ****Sport**** | **Training frequency** | ****Session duration**** | **Intervention duration** | ****Intervention components**** | **Knee injuries**  **(EG/CG)** |
| --- | --- | --- | --- | --- | --- | --- | --- | --- | --- | --- |
| Hewett et al., 1999 | NRCT | Ⅱ | EG：366  CG：463 | 14-18 | Soccer, volleyball, and basketball（W） | 3 sessions/week | 60–90 min | 6 weeks | Landing stabilization, plyometric training, balance training, agility training, and stretching | EG：2  CG：10 |
| Wedderkopp et al., 1999 | RCT | Ⅰ | EG：111  CG：126 | 16–18 | Handball（W） | Not reported (NR) | 10–15 min | One season, 10 months (August 1995 to May 1996) | Balance training | EG：2  CG：8 |
| Heidt et al., 2000 | NRCT | Ⅱ | EG：42  CG：258 | 14-18 | Soccer（W） | Mean frequency: 2.86 sessions/week | Not provided | 7 weeks | Plyometric training, agility training, and stretching | EG：3  CG：29 |
| Söderman M et al., 2000 | RCT | Ⅰ | EG：62  CG：78 | EG:20.4±4.6  CG:20.5±5.4 | Soccer（W） | First 30 days: daily (equivalent to 7 sessions/week);  thereafter: 3 sessions/week | 10–15 min | One outdoor season: April to October 1998 | Balance training | EG：8  CG：6 |
| Olsen et al., 2005 | RCT | Ⅰ | EG：958  CG：879 | EG：16.3±0.6  CG：16.2±0.6 | Handball (F/M) | Performed during each of the first 15 training sessions; thereafter once weekly | 15–20 min | One league season (8 months, September 2002 to April 2003) | Landing stabilization, plyometric training, balance training, agility training, and core training | EG：19  CG：38 |
| Petersen et al., 2005 | NRCT | Ⅱ | EG：134  CG：142 | Adults (exact values not reported) | Handball（W） | 3 sessions/week during preseason; 1 session/week during the season | 10 min/session during preseason (in-season duration not reported) | One season (start and end dates not reported, including 8 preseason weeks) | Landing stabilization, plyometric training, balance training, and agility training | EG：5  CG：9 |
| Emery et al.,2007 | RCT | Ⅰ | EG：494  CG：426 | EG：16 [13-18]  CG：16[12-18] | Basketball (F/M) | Before every training session (approximately 5 sessions/week) | Standard warm-up for 10 min; the intervention group additionally performed 5 min of balance training; plus 20 min of home-based balance-board training | 18 weeks (November 2004 to March 2005) | Balance training and stretching | EG：18  CG：13 |
| Soligard et al., 2008 | RCT | Ⅰ | EG：1055  CG：837 | 15.4±0.7 | Soccer（W） | 2–5 sessions/week (depending on the team’s weekly training frequency) | 20 min | One season, 8 months (March to October 2007) | Landing stabilization, plyometric training, balance training, agility training, stretching, and core training | EG：33  CG：47 |
| Steffen et al., 2008 | RCT | Ⅰ | EG：1073  CG：947 | 15.4±0.8 | Soccer（W） | During the first 15 training sessions: performed at every session; thereafter: once weekly for the remainder of the season。 Note: the article stated that many teams trained 1–2 times per week during the season. | 20 min | 8-month season (2005-03-01 to 2005-10-31) | Landing stabilization, plyometric training, balance training, agility training, and core training | EG：37  CG：30 |
| Gilchrist et al., 2008 | RCT | Ⅰ | EG：583  CG：852 | 19.88 | Soccer（W） | 3 sessions/week (although the prescribed frequency was 3 sessions/week, actual implementation varied across teams; the paper reported that the intervention teams used the program a mean of 25.8 times [range 12–37]) | Approximately 30 min (≤30 min) | 12 weeks | Landing stabilization, plyometric training, balance training, agility training, and stretching | EG：40  CG：58 |
| Emery & Meeuwisse, 2010 | RCT | Ⅰ | EG：380  CG：364 | 13–18 | Soccer (F/M) | Not quantifiable | 15 min | 20 weeks | Plyometric training, balance training, agility training, stretching, core training, and lower-limb strength training | EG：3  CG：8 |
| Kiani et al., 2010 | NRCT | Ⅱ | EG：777  CG：729 | EG 14.7 [12.7–18.6]  CG 15.0 [13.0–17.6] | Soccer（W） | Preseason: 2 sessions/week; in-season: 1 session/week | 20–25 min | 2007-02-01 to 2007-10-31 (including approximately 12 preseason weeks plus the season from 4/26 to 10/31) | Landing stabilization, plyometric training, balance training, agility training, stretching, and core training | EG：3  CG：13 |
| LaBella et al., 2011 | RCT | Ⅰ | EG：737  CG：755 | EG：16.19±1.53；  CG：16.22±1.06 | Soccer and basketball（W） | 3.3 sessions/week (mean) | 20 min | 13 weeks (mean) | Landing stabilization, plyometric training, balance training, agility training, and core training | EG：8  CG：17 |
| Longo et al., 2012 | RCT | Ⅰ | EG：80  CG：41 | EG：13.5±2.3  CG：15.2±4.6 | Basketball(M) | First month: training 6 times/week, and the intervention group performed the warm-up protocol in full at every training session; in the following months: training 3–4 times/week, but the warm-up protocol was performed at least twice weekly | 20 min | 9 months (August 2009 to April 2010) | Landing stabilization, plyometric training, balance training, agility training, stretching, and core training | EG：5  CG：2 |
| Waldén et al., 2012 | RCT | Ⅰ | EG：2479  CG：2085 | 12–17 | Soccer（W） | 2 sessions/week | 15 min plus 5 min of low-intensity jogging | 2009/4/1-2009/10/31 | Landing stabilization, plyometric training, balance training, and core training | EG：48  CG：44 |
| van Beijsterveldt et al., 2012 | RCT | Ⅰ | EG：223  CG：233 | EG :24.4±4.1  CG: 25.1±4.3 | Soccer(M) | ≥2 sessions/week; actual adherence: mean of approximately 1.3 sessions/week (team level) | 10–15 min | 33 weeks; one season (2009–2010, September to May of the following year) | Plyometric training, balance training, agility training, and core training | EG：24  CG：40 |
| Grooms et al., 2013 | NRCT | Ⅱ | EG：34  CG：30 | 20.1±2.0 | Soccer(M) | 5–6 sessions/week (recorded as “every training session/match,” equivalent to 5–6 sessions per week) | 20 min | 12 weeks | Plyometric training, balance training, agility training, stretching, and core training | EG：1  CG：1 |
| Owoeye et al.,2014 | RCT | Ⅰ | EG：212  CG：204 | EG：17.8 ±0.94  CG：17.5 ±1.10 | Soccer(M) | **≥2 sessions/week; actual adherence was 1.6 sessions/week** | 20 min | **6 months (September 2012 to February 2013)** | Plyometric training, balance training, agility training, stretching, and core training | EG：12  CG：21 |
| Finch et al., 2016 | RCT | Ⅰ | EG：679  CG：885 | ≥18 | Rugby(M) | 2 sessions/week | 20 min | 26 weeks (8 preseason weeks + 18 in-season weeks) | Landing stabilization, plyometric training, balance training, agility training, and core training | EG：32  CG：60 |
| Silvers-Granelli et al., 2017 | RCT | Ⅰ | EG：675  CG：850 | EG：20±2  CG：21±1 | Soccer(M) | 2–3 sessions/week; actual adherence was 2.19 sessions/week | 15–20 min | One season, August 2012 to December 2012 | Plyometric training, balance training, agility training, stretching, and core training | EG：34  CG：102 |
| Bonato et al., 2018 | RCT | Ⅰ | EG：86  CG：74 | EG：20±2；  CG：20±1； | Basketball（W） | 4 sessions/week | 30 min | 2015-10-04 to 2016-04-10 | Landing stabilization, plyometric training, balance training, agility training, stretching, and core training | EG：2  CG：19 |
| Barber Foss et al., 2018 | RCT | Ⅰ | EG：259  CG：215 | 14.0 ± 1.7 | Basketball, soccer, and volleyball（W） | 3 sessions/week during preseason; 2 sessions/week during the season | 20–25 min during preseason; 10–15 min during the season | NA (start and end dates not reported in the original article) | Landing stabilization, plyometric training, balance training, and core training | EG：60  CG：72 |
| Slauterbeck et al., 2019 | RCT | Ⅰ | EG：1825  CG：1786 | 14-18 | American football (M), soccer (M/F), basketball (M/F), and hockey (M/F) | 1.45 sessions/week (mean) | 15–20 min | 12 weeks | Balance training, stretching, and core training | EG：35  CG：41 |
| Bhat & Sreedhar, 2019 | NRCT | Ⅱ | EG：23  CG：21 | EG:22.22  CG:22.05 | Basketball(M) | 4 sessions/week | Not reported | 8 weeks | Agility training | EG：1  CG：3 |
| Yarsiasat et al., 2019 | RCT | Ⅰ | EG：26  CG：26 | EG 15.50±1.10  CG: 15.19±1.26 | Sepak takraw（W） | 3 sessions/week | 20 min | 8 weeks | Plyometric training, agility training, stretching, and core training | EG：3  CG：9 |
| Akerlund et al., 2020 | RCT | Ⅰ | EG：301  CG：170 | EG：13.6±1.1  CG：13.2±1.3 | Floorball (F/M) | ≥2 sessions/week | 15–20 min | 26 weeks | Landing stabilization, plyometric training, balance training, and core training | EG：87  CG：57 |
| Lopes et al., 2020 | RCT | Ⅰ | EG：31  CG：34 | EG: 27.7±5.0  CG: 25.8±4.9 | Futsal(M) | 2 sessions/week | 20 min | 20 weeks | Plyometric training, balance training, agility training, stretching, and core training | EG：4  CG：6 |
| Hilska et al.,2021 | RCT | Ⅰ | EG：673  CG：730 | 9-14 | Soccer (F/M) | 2–3 sessions/week (actual adherence: 1.7 sessions/week) | 20–25 min/session; actual recorded duration was 25 min/session | 20 weeks (January to June 2015) | Plyometric training, balance training, agility training, and core training | EG：61  CG：70 |
| Sommerfield et al., 2021 | NRCT | Ⅱ | EG：53  CG：50 | EG: 13.9±0.6  CG: 14.1±0.5 | Netball, soccer, field hockey, lacrosse, swimming, track and field, badminton, and rowing（W） | Unclear | 21.7 min/session (mean) | 23 weeks | Landing stabilization, plyometric training, balance training, agility training, and core training | EG：31  CG：12 |
| Obertinca et al., 2024 | RCT | Ⅰ | EG：524  CG：503 | EG: 15.2±1.6  CG:15.3±1.6 | Soccer(M) | Planned implementation: ≥2 sessions/week; actual adherence: 2.2 sessions/week | 15–20 min/session | One season (August 2021 to May 2022) | Plyometric training, balance training, agility training, and core training | EG：26  CG：36 |

**Note:** Age is presented in the order of **EG/CG**. A single value indicates the **mean**. **a–b** indicates the **age range**. **x ± y** indicates the **mean ± standard deviation**. **x [a–b]** indicates the **median [a–b]**. **RCT** = randomized controlled trial; **NRCT** = non-randomized controlled trial. **F** = female; **M** = male.

**Table 2 Injury definitions, surveillance characteristics, exposure information, and injury-counting approaches of included studies**

| **Study** | **Outcome metric reported** | **Exposure denominator retained** | **Injury-counting approach** | **Recurrent injuries** |
| --- | --- | --- | --- | --- |
| Hewett et al. (1999) | Serious knee injuries; knee injury incidence reported per 1000 athlete-exposures; noncontact knee injuries and ACL injuries also reported. | Yes. Athlete-exposures were reported and defined as one athlete participating in one practice or match. | Serious knee injury events during sport participation were counted. Only primary injuries were included in the main analysis. | Reinjuries were tabulated but not reported; therefore recurrent injuries were not included in the reported main outcome. |
| Wedderkopp et al. (1999) | Overall injuries, traumatic injuries, overuse injuries, and injury incidence per 1000 hours of practice and match; injury type and location were reported. | Yes. Practice exposure time and match-related injury incidence were reported; exposure was expressed as hours of practice and match participation. | Injury occurrences were counted if they occurred during scheduled games or practices and caused missed participation or substantial discomfort. Injuries were classified as minor, moderate, major, traumatic, or overuse. | Previous/same-type injuries were recorded in the injury questionnaire, but recurrent injuries were not clearly separated from the main outcome. |
| Heidt et al. (2000) | Time-loss lower-extremity injuries; knee injuries by anatomical diagnosis; percentage of injured athletes; ACL injury percentage also reported. | No. Practice/game setting was recorded, but total athlete-exposure time or athlete-exposures were not reported. | Injury events causing the athlete to miss a game or practice were counted. Both total injuries and injured athletes were reported. | Not clearly reported. Five athletes sustained two injuries, indicating that multiple injury events were counted, but recurrent injuries were not separately identified. |
| Söderman et al. (2000) | Traumatic lower-extremity injuries; number of injured players; number, type, and incidence rate of traumatic injuries; ACL injuries also reported. | Yes. Injury incidence was reported per 1000 hours for soccer exposure, practice, and games. | Traumatic lower-extremity injury events and injured players were reported. Time to first injury was also analyzed. | Included/reported. Reinjuries accounted for 36% of injuries in the intervention group and 45% in the control group. |
| Olsen et al. (2005) | Acute knee or ankle injuries as the primary outcome; acute knee injuries reported separately; injured-player risk and injury incidence per 1000 player-hours were reported. | Yes. Player-hours were reported separately for matches and training. | Intention-to-treat analysis based on the number of injured players; injury incidence was also calculated using player-hours. | Not specifically reported as recurrent vs first injuries. All eligible acute injuries during follow-up were included. |
| Petersen et al. (2005) | All injuries, lower-extremity injuries, knee injuries, ankle injuries, and ACL injuries; injury incidence was reported per 1000 h of handball exposure. | Yes. Exposure time was retained and expressed as hours of handball participation, with incidence reported per 1000 h. | Injury events were documented weekly over one season. Knee injuries and ACL injuries were reported as the main knee-related outcomes. | Not clearly reported. The study did not explicitly distinguish first injuries from recurrent injuries in the main outcome. |
| Emery et al. (2007) | All injuries, acute-onset injuries, lower-extremity injuries, and ankle sprain injuries; injury rates were reported per 1000 player-hours with cluster-adjusted relative risks. | Yes. Total player exposure hours were recorded for the control and training groups using weekly exposure sheets. | Injury events were counted if they occurred during basketball and required medical attention, removal from the current session, or missing a subsequent session. | Not separately reported. Multiple or recurrent injuries were not clearly separated in the main outcome. |
| Soligard et al. (2008) | Lower-extremity injuries as the primary outcome; overall injuries, acute injuries, overuse injuries, severe injuries, and knee injuries were also reported; effect estimates were reported as rate ratios. | Yes. Match exposure and training exposure were retained and expressed as player-hours. | Injuries were counted if they occurred during scheduled match or training and caused the player to be unable to fully participate in the next match or training session. | Included and defined. Re-injury was defined as an injury of the same type and location sustained previously. |
| Steffen et al. (2008) | Overall injuries, acute injuries, overuse injuries, injury location including knee injuries, ACL injuries, contact/non-contact injuries, severity, and re-injuries; incidence was reported per 1000 h of exposure with rate ratios. | Yes. Match and training exposure were retained as player-hours, but exposure was collected on a team basis rather than at the individual-player level. | Time-loss injuries were counted if the player could not fully participate in the next match or training session. Acute, overuse, location, mechanism, and severity were classified. | Included and defined. Recurrent injuries were defined as the same type and site after return to full participation; re-injuries were also reported separately. |
| Gilchrist et al. (2008) | Knee injuries, ACL injuries, and noncontact ACL injuries; ACL injury rates were reported per 1000 athlete-exposures, with rate-ratio comparisons. | Yes. Athlete-exposures from games and practices were retained and used to calculate injury rates per 1000 athlete-exposures. | Knee injuries were counted if they occurred during game, practice, or conditioning, required medical care, and caused at least one missed day of training or play. ACL injuries were analyzed separately, including noncontact ACL injuries. | Included/partly reported. Athletes with a history of ACL injury were analyzed, and subsequent ACL injuries in this subgroup were reported; recurrent injuries were not separated from the main overall knee-injury count. |
| Emery & Meeuwisse (2010) | All soccer injuries, acute-onset injuries, lower-extremity injuries, ankle sprain injuries, and knee sprain injuries; injury rates were reported per 1000 player-hours, with adjusted incidence rate ratios. | Yes. Exposure was recorded as player-hours for games and practices using weekly exposure sheets. | Injuries were counted if they occurred during soccer and required medical attention, removal from a session, or time loss from full soccer participation. | Not separately reported. Previous injury was considered as a covariate, but recurrent injuries were not clearly separated in the outcome count. |
| Kiani et al. (2010) | New acute knee injuries diagnosed by physicians; noncontact knee injuries and ACL injuries were also reported; incidence rates were reported per 1000 player-hours. | Yes. Total soccer exposure was retained as player-hours, including both training exposure and match exposure. | Only new acute knee injuries occurring between February 1 and October 31, 2007 were counted as the main outcome. These were defined as traumatic knee injuries requiring medical care, with diagnoses confirmed by orthopedic physicians and medical records. | Excluded from the main outcome. Two ACL reinjuries and one knee distortion without physician diagnosis were disregarded as outcomes; a sensitivity analysis including these cases did not change the interpretation. |
| LaBella et al. (2011) | Lower-extremity injuries, acute-onset noncontact lower-extremity injuries, noncontact knee sprains, and noncontact ACL sprains; rates were reported per 1000 athlete-exposures, with adjusted incidence rate ratios. | Yes. Athlete-exposures were retained and defined as one athlete participating in all or part of a practice or game. | Injuries were counted if they occurred during practice or game and resulted in time loss from a practice or game. Injured athletes were interviewed, and ACL injuries were verified by MRI reports or operative notes. | Not separately reported. Previous sports-related injury was collected, but recurrent injuries were not clearly separated from the main outcome. |
| Longo et al. (2012) | Overall injuries as the primary outcome; lower-extremity injuries, knee injuries, ankle injuries, acute injuries, overuse injuries, and severe injuries were also reported; injury rates were reported per 1000 athlete-exposures. | Yes. Exposure was retained as athlete-exposures from training and matches. | Injury events were recorded through daily injury reports compiled by coaches, including injury location, type, and exposure type. Injuries were classified according to international consensus-based injury classification. | Not clearly reported. Recurrent injuries were not separately defined or reported in the main outcome. |
| Waldén et al. (2012) | ACL injury as the primary outcome; severe knee injury and any acute knee injury as secondary outcomes; rate ratios were estimated using Cox regression with robust standard errors. | Yes. Individual playing time was recorded in minutes for each player for training and match exposure and converted into playing hours. | Acute knee injuries were counted if they occurred during football training or match play, had sudden onset, and caused inability to fully participate in future training or match play. Contusions were excluded. | Included for ACL outcome. ACL injury was defined as a first or recurrent partial or total rupture, either isolated or with concomitant knee injuries. |
| van Beijsterveldt et al. (2012) | All soccer injuries as the primary outcome; injury incidence per 1000 h of soccer participation; injury location, including knee injuries, injury mechanism, severity, and recurrence were also reported. | Yes. Exposure was retained as total hours of soccer participation, including practice and match exposure. | Injuries were counted as any physical complaint sustained during soccer match or practice, irrespective of the need for medical attention or time loss. Injury data were recorded using a web-based injury registration system. | Included and defined. Recurrent injury was defined as an injury of the same type and at the same site as a previous injury after return to full participation. Analyses were performed both without reinjuries and with first-time plus recurrent injuries. |
| Grooms et al. (2013) | Lower-extremity injury risk and time lost to lower-extremity injury; injury incidence was reported per 1000 athlete-exposures and also per 1000 training/match hours; specific injury diagnoses included knee MCL sprain and knee meniscus injury. | Yes. Athlete-exposures were retained for practices and games; training and match exposure hours were also reported. | Time-loss injuries were counted if they required medical attention and caused loss of at least 1 day of activity. Injuries were tracked daily by the same certified athletic trainer. | Not separately reported. Recurrent injuries were not clearly defined or separated in the main outcome. |
| Owoeye et al. (2014) | All injuries, match injuries, training injuries, lower-extremity injuries, and injuries by body location, aetiology, mechanism, and severity; injury incidence was reported per 1000 h. | Yes. Exposure was retained using team-level records of matches, training sessions, and player participation. | Time-loss injuries were counted if they caused players to be unable to fully participate in subsequent football training sessions or matches. Injuries were assessed weekly by study physiotherapists. | Not separately reported. Recurrent injuries were not clearly defined or separated in the main outcome. |
| Finch et al. (2016) | All game-related injuries, lower-limb injuries, and knee injuries; injury incidence rates were reported per 1000 game hours, with cluster-adjusted injury rate ratios. | Yes. Game exposure was retained as hours of game participation. Training participation was recorded for intervention delivery, but the reported injury analysis focused on game-related injuries. | Game injuries were counted if they caused the player to seek medical attention on or off the field or to leave the field of play. Injury details were recorded by primary data collectors and confirmed where possible. | Not clearly reported. Recurrent injuries were not separately defined or reported in the main outcome. |
| Silvers-Granelli et al. (2017) | Knee injuries and ACL injuries, including ACL injuries by game/practice setting, player position, division level, and field type; injury rates were reported per 1000 athletic exposures, with relative risks. | Yes. Athletic exposures were retained and entered weekly by certified athletic trainers. Injury rates were calculated per 1000 athletic exposures. | Injuries, including ACL injuries, were entered weekly by each team’s certified athletic trainer, with mechanism of injury and return-to-play date recorded. Data accuracy was confirmed against each institution’s internal injury database. | Not separately reported. Previous knee/ACL injury history was considered in the background and data collection context, but recurrent injuries were not clearly separated from the main outcome. |
| Bonato et al. (2018) | All injuries, lower-limb injuries, knee sprains, ankle sprains, ACL lesions, acute injuries, overuse pain, and severe injuries; epidemiologic incidence proportion and incidence rate were reported. | Yes. Exposure was retained as training and match participation time, and incidence rate was calculated as injuries per 1000 h of exposure. | Injuries were counted if they occurred during scheduled training or match play and caused the player to be unable to fully participate in the next training session or match. Injury type, location, and severity were classified. | Partly included. ACL injury was defined as a first or recurrent partial or total rupture, but recurrent injuries were not separately reported in the main outcome. |
| Barber Foss et al. (2018) | Overall sport-related injuries, sport-specific injuries, knee injuries, ankle injuries, and ACL injuries; injury rates were reported per 1000 athlete-exposures. | Yes. Athlete-exposures were retained and defined as one athlete participating in one coach-directed session, including game or practice. | Injuries were counted if they caused cessation of participation in the current session, cessation on the next day, or involved fracture, dental injury, or mild brain injury. Athletes were evaluated weekly by athletic trainers. | Multiple injuries per athlete were included statistically, but recurrent injuries were not clearly defined or separately reported. |
| Slauterbeck et al. (2019) | Lower-extremity injuries as the main outcome; knee and ankle injury locations were also reported; injury incidence was reported per 1000 athlete-exposures with cluster-adjusted comparisons. | Yes. Athlete-exposures were recorded and used to calculate lower-extremity injury incidence per 1000 athlete-exposures. | Injuries were counted if the athlete was unable to fully participate in the subsequent practice or game. Injury location, type, severity, time loss, and setting were recorded by certified athletic trainers. | Included/reported descriptively. Most injuries were new rather than recurrent, but recurrent injuries were not used as a separate main outcome. |
| Bhat & Sreedhar (2019) | Ankle and knee injuries as the primary outcome; lower-limb injuries and all injuries were also reported, with injury counts by body region and type. | Partly/unclear. The study referred to training and match injuries and stated that exposures were calculated, but detailed exposure denominators were not clearly reported. | Injured players and injury events were counted over the 8-week intervention period. Injuries were classified by body region and by acute or overuse type. | Not clearly reported. Recurrent injuries were not separately defined or distinguished from first injuries. |
| Yarsiasat et al. (2019) | Knee injury incidence among adolescent female Sepak takraw players; injury characteristics included ACL tear, chondromalacia patella, muscle soreness/strain, player position, cause, and mechanism; incidence was reported per 100,000 athlete-exposures. | Yes. Athlete-exposures were retained and injury incidence was reported per 100,000 athlete-exposures. | Knee injuries were assessed prospectively after the 8-week training period, with injury number and characteristics measured during 6-month follow-up by orthopedic assessment. | Not clearly reported. Recurrent injuries were not separately defined or reported. |
| Åkerlund et al. (2020) | All floorball injuries as the primary outcome; acute injuries, overuse injuries, substantial injuries, time-loss injuries, medical-attention injuries, lower-limb injuries, and knee injuries were also reported; injury incidence was reported per 1000 h of floorball exposure. | Yes. Exposure was retained as hours of floorball training and match play, based on weekly player reports and coach-reported playing time. | Injuries were counted as unique injury events, including both new and recurrent injuries. Weekly OSTRC-based player reports were used, with additional follow-up for time-loss and medical-attention injuries. | Included and clearly defined. Reinjury was defined as an injury of the same type and same site as an index injury in the current or preceding season. Recurrent injuries were included in the injury-event count after at least 1 week of full participation without health problems. |
| Lopes et al. (2020) | Overall injuries, acute injuries, lower-limb injuries, training injuries, time-loss injuries, injury severity, and injury type/location were reported; injury incidence was reported per 1000 player-hours. | Yes. Match and training exposure were retained as player-hours. The intervention group accumulated 3977 h of futsal exposure and the control group 3303 h. | Injuries were recorded during the regular futsal season using football injury-surveillance consensus procedures. Injury categories included acute injuries, lower-limb injuries, training/match injuries, and time-loss injuries. | Not clearly reported. Recurrent injuries were not separately defined or distinguished from first injuries in the main outcome. |
| Hilska et al. (2021) | Acute lower-extremity soccer injuries as the primary outcome; acute noncontact lower-extremity injuries as the secondary outcome; ankle, joint/ligament, and time-loss subgroups were also reported; incidence was reported per 1000 h of exposure with IRRs. | Yes. Exposure was retained as soccer practice and game hours. Practice exposure was based on team practice data, and game exposure was calculated from game duration and number of players on the field. | Injuries were counted as soccer-related acute lower-extremity injuries. Weekly SMS surveillance was used, and each new injury was followed up by a blinded physiotherapist using a structured questionnaire. | Not clearly reported. Previous injury was discussed, but recurrent injuries were not clearly separated from first injuries in the main outcome. |
| Sommerfield et al. (2021) | Any injury rate, acute injuries, gradual-onset/overuse injuries, and training versus competition injury rates were reported; injury rates were expressed per 1000 h of exposure. | Yes, but self-reported. Weekly training and competition exposure were collected using an online questionnaire. | Injuries were counted as physical problems affecting training or competition in the previous week. New injury cases were determined from questionnaire responses and weekly follow-up interviews, and injuries were classified as acute or gradual-onset/overuse. | Not clearly reported. Recurrent injuries were not separately defined or reported in the main outcome. |
| Obërtinca et al. (2024) | Overall football-related injuries as the primary outcome; secondary outcomes included lower-limb region-specific injuries, including hip/groin, thigh, knee, lower leg, ankle and foot injuries, and injury severity; IRRs were reported. | Yes. Exposure was retained as team exposure hours. The original plan was individual exposure, but the study changed to team-level exposure reporting during data collection. | New football-related injuries were reported weekly by coaches or team physiotherapists. Blinded physiotherapists contacted injured players or parents to collect detailed injury information using a standardized injury registration form. | Not clearly reported. New injuries were collected prospectively, but recurrent injuries were not clearly separated from first injuries in the main outcome. |

**Note:** Exposure information was retained during data extraction to characterize outcome reporting across studies. Effect-size selection and conversion procedures are described in the Statistical Analysis section.

**Table 3 Cluster-adjustment categories**

| **Category** | **Studies** | **Handling in the main analysis** |
| --- | --- | --- |
| A | Olsen 2005；Soligard 2008；Emery & Meeuwisse 2010；Kiani 2010；LaBella 2011；Waldén 2012；Finch 2016；Åkerlund 2020；Obërtinca 2024 | Original knee-specific cluster-adjusted RR, IRR, or rate ratio was used where applicable. |
| B | Emery 2007；Slauterbeck 2019 | Cluster-adjusted estimates were available for broader injury outcomes, but no knee-specific cluster-adjusted estimate was available; event-count-based ORs for knee injuries were used in the main analysis, and these studies were excluded in the sensitivity analysis. |
| C | Hewett 1999；Wedderkopp 1999；Söderman 2000；Petersen 2005；Steffen 2008；Gilchrist 2008；Longo 2012；van Beijsterveldt 2012；Owoeye 2014；Silvers-Granelli 2017；Bonato 2018；Barber Foss 2018；Lopes 2020；Hilska 2021 | Suspected or actual team-level or cluster-level design, but no knee-specific cluster-adjusted estimate was available; event-count-based ORs for knee injuries were used in the main analysis, and these studies were excluded in the sensitivity analysis. |
| D | Heidt 2000；Grooms 2013；Bhat & Sreedhar 2019；Yarsiasat 2019；Sommerfield 2021 | Not applicable; the study did not involve cluster randomization or cluster-level allocation, and event-count-based ORs were used. |

**Note:** Category A = studies with knee-specific cluster-adjusted estimates available and used where applicable; Category B = studies with cluster-adjusted estimates for broader injury outcomes but without knee-specific cluster-adjusted estimates; Category C = cluster-randomized or cluster-level studies without available knee-specific cluster-adjusted estimates, for which event-count-based estimates were used in the main analysis; Category D = studies for which cluster adjustment was not applicable. Studies in Categories B and C were excluded in the sensitivity analysis evaluating the influence of unclear or unadjusted cluster adjustment.

**Table 4 Cluster-adjustment status, availability of knee-specific adjusted estimates, and effect estimate used in the primary meta-analysis**

| **Study** | **Unit of allocation** | **Category** | **Knee-specific cluster-adjusted estimate** |
| --- | --- | --- | --- |
| Hewett et al. (1999) | Team-level self-selection | **C** | — |
| Wedderkopp et al. (1999) | Team | C | — |
| Heidt et al. (2000) | Individual athlete | D | — |
| Söderman et al. (2000) | Team | **C** | — |
| Olsen et al. (2005) | Club | A | RR = 0.45，95% CI 0.25–0.81，P = 0.007； |
| Petersen et al. (2005) | Matched teams | C | — |
| Emery et al. (2007) | School | B | — |
| Soligard et al. (2008) | Club | A | Rate ratio = 0.62，95% CI 0.36–1.05，P = 0.079；ICC = 0.028，inflation factor = 1.54 |
| Steffen et al. (2008) | Team/club | C | — |
| Gilchrist et al. (2008) | Team | C | — |
| Emery & Meeuwisse (2010) | Club/team | A | IRR = 0.38, 95% CI 0.08–1.75, p = 0.232 |
| Kiani et al. (2010) | Region/team-level allocation | A | RR = 0.17, 95% CI 0.04–0.64；p value not clearly reported |
| LaBella et al. (2011) | School | A | IRR = 0.30, 95% CI 0.10–0.86, p = 0.03； |
| Longo et al. (2012) | Team | C | — |
| Waldén et al. (2012) | Club | A | RR = 0.92，95% CI 0.61–1.40，P = 0.71 |
| van Beijsterveldt et al. (2012) | Team/competition cluster | C | — |
| Grooms et al. (2013) | Season cohort | D | — |
| Owoeye et al. (2014) | Team | C | — |
| Finch et al (2016) | Club | A | IRR = 0.50, 95% CI 0.24–1.05, p = 0.07 |
| Silvers-Granelli et al. (2017) | Team/institution | C | — |
| Bonato et al. (2018) | Team/club | C | — |
| Barber Foss et al. (2018) | Team | C | — |
| Slauterbeck et al. (2019) | School | B | — |
| Bhat & Sreedhar (2019) | Individual athlete | D | — |
| Yarsiasat et al. (2019) | Individual athlete | D | — |
| Åkerlund et al. (2020) | Club cluster | A | IRR = 0.76, 95% CI 0.54–1.08, p = 0.127 |
| Lopes et al. (2020) | Team | **C** | — |
| Hilska et al. (2021) | Club/city cluster | C | — |
| Sommerfield et al. (2021) | School-selected group | D | — |
| Obërtinca et al. (2024) | Club | A | IRR = 0.71, 95% CI 0.43–1.18, p = 0.193 |

**Note:** Category A = studies with knee-specific cluster-adjusted estimates available and used where applicable; Category B = studies with cluster-adjusted estimates for broader injury outcomes but without knee-specific cluster-adjusted estimates; Category C = cluster-randomized or cluster-level studies without available knee-specific cluster-adjusted estimates, for which event-count-based estimates were used in the main analysis; Category D = studies for which cluster adjustment was not applicable. Studies in Categories B and C were excluded in the sensitivity analysis evaluating the influence of unclear or unadjusted cluster adjustment. For studies without available knee-specific adjusted estimates, event-count-based ORs were calculated from knee-injury counts and used in the primary analysis. For studies with available knee-specific adjusted estimates, the reported RR, IRR, or rate ratio was extracted and log-transformed for pooling.

**Table 5 Study-level classification of standardized versus non-standardized NMT programs**

| **Study** | **Fixed and clearly defined structure** | **Clearly specified dose** | **Progression or difficulty levels** | **Quality-of-execution or technical standards** | **Protocol reproducibility** | **Total criteria met** | **Final classification** |
| --- | --- | --- | --- | --- | --- | --- | --- |
| Hewett et al., 1999 | Yes | Yes | Yes | Yes | Yes | 5/5 | Standardized |
| Wedderkopp et al., 1999 | No | Yes | No | No | No | 1/5 | Non-standardized |
| Heidt et al., 2000 | No | Yes | Yes | Yes | No | 3/5 | Non-standardized |
| Soderman M et al., 2000 | Yes | Yes | Yes | Yes | Yes | 5/5 | Standardized |
| Olsen et al., 2005 | Yes | Yes | Yes | Yes | Yes | 5/5 | Standardized |
| Petersen et al., 2005 | Yes | Yes | Yes | Yes | Yes | 5/5 | Standardized |
| Emery et al.,2007 | Yes | Yes | Yes | No | No | 3/5 | Non-standardized |
| Soligard et al., 2008 | Yes | Yes | Yes | Yes | Yes | 5/5 | Standardized |
| Steffen et al., 2008 | Yes | Yes | No | Yes | Yes | 4/5 | Standardized |
| Gilchrist et al., 2008 | Yes | Yes | No | Yes | No | 3/5 | Non-standardized |
| Emery & Meeuwisse, 2010 | Yes | No | No | No | No | 1/5 | Non-standardized |
| Kiani et al., 2010 | Yes | Yes | No | Yes | Yes | 4/5 | Standardized |
| LaBella et al., 2011 | Yes | Yes | Yes | Yes | Yes | 5/5 | Standardized |
| Longo et al., 2012 | Yes | Yes | Yes | Yes | Yes | 5/5 | Standardized |
| Waldén et al., 2012 | Yes | Yes | Yes | Yes | Yes | 5/5 | Standardized |
| van Beijsterveldt et al., 2012 | Yes | Yes | No | Yes | Yes | 4/5 | Standardized |
| Grooms et al., 2013 | Yes | Yes | Yes | Yes | Yes | 5/5 | Standardized |
| Owoeye et al., 2014 | Yes | Yes | Yes | Yes | Yes | 5/5 | Standardized |
| Finch et al., 2016 | Yes | Yes | Yes | No | Yes | 4/5 | Standardized |
| Silvers-Granelli et al., 2017 | Yes | Yes | Yes | Yes | Yes | 5/5 | Standardized |
| Bonato et al., 2018 | Yes | Yes | Yes | Yes | Yes | 5/5 | Standardized |
| Barber Foss et al., 2018 | Yes | Yes | Yes | Yes | Yes | 5/5 | Standardized |
| Slauterbeck et al., 2019 | Yes | No | Yes | Yes | No | 3/5 | Non-standardized |
| Bhat & Sreedhar, 2019 | Yes | Yes | Yes | No | No | 3/5 | Non-standardized |
| Yarsiasat et al., 2019 | Yes | Yes | No | No | No | 2/5 | Non-standardized |
| Akerlund et al., 2020 | Yes | Yes | Yes | Yes | Yes | 5/5 | Standardized |
| Lopes et al., 2020 | Yes | No | Yes | No | Yes | 3/5 | Non-standardized |
| Hilska et al.,2021 | Yes | No | No | No | Yes | 2/5 | Non-standardized |
| Sommerfield et al., 2021 | Yes | No | Yes | Yes | No | 3/5 | Non-standardized |
| Obertinca et al., 2024 | Yes | Yes | Yes | Yes | Yes | 5/5 | Standardized |

**Table 6 List of included studies**

| **No.** | **Author/Year** | **Corresponding reference** |
| --- | --- | --- |
| 1 | Hewett et al., 1999 | Hewett TE, Lindenfeld TN, Riccobene JV, Noyes FR. The effect of neuromuscular training on the incidence of knee injury in female athletes. A prospective study. Am J Sports Med. 1999;27(6):699–706. |
| 2 | Wedderkopp et al., 1999 | Wedderkopp N, Kaltoft M, Lundgaard B, Rosendahl M, Frøberg K. Prevention of injuries in young female players in European team handball. A prospective intervention study. Scand J Med Sci Sports. 1999;9(1):41–47. |
| 3 | Heidt et al., 2000 | Heidt RS Jr, Sweeterman LM, Carlonas RL, Traub JA, Tekulve FX. Avoidance of soccer injuries with preseason conditioning. Am J Sports Med. 2000;28(5):659–662. |
| 4 | Söderman M et al., 2000 | Söderman K, Werner S, Pietilä T, Engström B, Alfredson H. Balance board training: prevention of traumatic injuries of the lower extremities in female soccer players. A prospective randomized intervention study. Knee Surg Sports Traumatol Arthrosc. 2000;8(6):356–363. |
| 5 | Olsen et al., 2005 | Olsen OE, Myklebust G, Engebretsen L, Holme I, Bahr R. Exercises to prevent lower limb injuries in youth sports: cluster randomised controlled trial. BMJ. 2005;330:449. |
| 6 | Petersen et al., 2005 | Petersen W, Braun C, Bock W, Schmidt K, Weimann A, Drescher W, et al. A controlled prospective case control study of a prevention training program in female team handball players: the German experience. Arch Orthop Trauma Surg. 2005;125(9):614–621. |
| 7 | Emery et al.,2007 | Emery CA, Rose MS, McAllister JR, Meeuwisse WH. A prevention strategy to reduce the incidence of injury in high school basketball: a cluster randomized controlled trial. Clin J Sport Med. 2007;17(1):17–24. |
| 8 | Soligard et al., 2008 | Soligard T, Myklebust G, Steffen K, Holme I, Silvers H, Bizzini M, et al. Comprehensive warm-up programme to prevent injuries in young female footballers: cluster randomised controlled trial. BMJ. 2008;337:a2469. |
| 9 | Steffen et al., 2008 | Steffen K, Myklebust G, Olsen OE, Holme I, Bahr R. Preventing injuries in female youth football—a cluster-randomized controlled trial. Scand J Med Sci Sports. 2008;18(5):605–614. |
| 10 | Gilchrist et al., 2008 | Gilchrist J, Mandelbaum BR, Melancon H, Ryan GW, Silvers HJ, Griffin LY, et al. A randomized controlled trial to prevent noncontact anterior cruciate ligament injury in female collegiate soccer players. Am J Sports Med. 2008;36(8):1476–1483. |
| 11 | Emery & Meeuwisse, 2010 | Emery CA, Meeuwisse WH. The effectiveness of a neuromuscular prevention strategy to reduce injuries in youth soccer: a cluster-randomised controlled trial. Br J Sports Med. 2010;44(8):555–562. |
| 12 | Kiani et al., 2010 | Kiani A, Hellquist E, Ahlqvist K, Gedeborg R, Michaëlsson K, Byberg L. Prevention of soccer-related knee injuries in teenaged girls. Arch Intern Med. 2010;170(1):43–49. |
| 13 | LaBella et al., 2011 | LaBella CR, Huxford MR, Grissom J, Kim KY, Peng J, Christoffel KK. Effect of neuromuscular warm-up on injuries in female soccer and basketball athletes in urban public high schools: cluster randomized controlled trial. Arch Pediatr Adolesc Med. 2011;165(11):1033–1040. |
| 14 | Longo et al., 2012 | Longo UG, Loppini M, Berton A, Marinozzi A, Maffulli N, Denaro V. The FIFA 11+ program is effective in preventing injuries in elite male basketball players: a cluster randomized controlled trial. Am J Sports Med. 2012;40(5):996–1005. |
| 15 | Waldén et al., 2012 | Waldén M, Atroshi I, Magnusson H, Wagner P, Hägglund M. Prevention of acute knee injuries in adolescent female football players: cluster randomised controlled trial. BMJ. 2012;344:e3042. |
| 16 | van Beijsterveldt et al., 2012 | van Beijsterveldt AMC, van de Port IGL, Krist MR, Schmikli SL, Stubbe JH, Frederiks JE, et al. Effectiveness of an injury prevention programme for adult male amateur soccer players: a cluster-randomised controlled trial. Br J Sports Med. 2012;46(16):1114–1118. |
| 17 | Grooms et al., 2013 | Grooms DR, Palmer T, Onate JA, Myer GD, Grindstaff T. Soccer-specific warm-up and lower extremity injury rates in collegiate male soccer players. J Athl Train. 2013;48(6):782-89 |
| 18 | Owoeye et al.,2014 | Owoeye OB, Akinbo SR, Tella BA, Olawale OA. Efficacy of the FIFA 11+ Warm-Up Programme in Male Youth Football: A Cluster Randomised Controlled Trial. J Sports Sci Med. 2014;13(2):321-8 |
| 19 | Finch et al., 2016 | Finch CF, Twomey DM, Fortington LV, et al. Preventing Australian football injuries with a targeted neuromuscular control exercise programme: comparative injury rates from a training intervention delivered in a clustered randomised controlled trial.Inj Prev. 2016;22(2):123-28 |
| 20 | Silvers-Granelli et al., 2017 | Silvers-Granelli HJ, Bizzini M, Arundale A, Mandelbaum BR, Snyder-Mackler L. Does the FIFA 11+ Injury Prevention Program Reduce the Incidence of ACL Injury in Male Soccer Players? Clin Orthop Relat Res.2017;475(10):2447‐55 |
| 21 | Bonato et al., 2018 | Bonato M, Benis R, La Torre A. Neuromuscular training reduces lower limb injuries in elite female basketball players. A cluster randomized controlled trial. Scand J Med Sci Sports 2018;28(4):1451-60 |
| 22 | Barber Foss et al., 2018 | Barber Foss KD, Thomas S, Khoury JC, Myer GD, Hewett TE. A school-based neuromuscular training program and sport-related injury incidence: a prospective randomized controlled clinical trial. J Athl Train. 2018;53(1):20–28. |
| 23 | Slauterbeck et al., 2019 | Slauterbeck JR, Choquette R, Tourville TW, et al. Implementation of the FIFA 11+ Injury Prevention Program by High School Athletic Teams Did Not Reduce Lower Extremity Injuries: A Cluster Randomized Controlled Trial. Am J Sports Med. 2019;47(12):2844-52 |
| 24 | Bhat & Sreedhar, 2019 | Bhat NA, Sreedhar K. A study on the effect of eight weeks exercise training protocol to prevent ankle and knee injuries among basketball players. Indian J Public Health Res Dev. 2019;10(5):187-91 |
| 25 | Yarsiasat et al., 2019 | Yarsiasat J, Sumannont S, Manimmanakorn N, Srilamarth S. Effectiveness of the Prevent Injury Enhance Performance (PEP) training program in reducing injury incidence rates among adolescent female Sepak takraw players: a randomised controlled trial. J Med Assoc Thai. 2019;102:98-105 |
| 26 | Akerlund et al., 2020 | Åkerlund I, Waldén M, Sonesson S, Hägglund M. Forty-five per cent lower acute injury incidence but no effect on overuse injury prevalence in youth floorball players (aged 12-17 years) who used an injury prevention exercise programme: two-armed parallel-group cluster randomised controlled trial. Br J Sports Med 2020;54(17):1028-35 |
| 27 | Lopes et al., 2020 | Lopes M, Simões D, Costa R, Oliveira J, Ribeiro F. Effects of the FIFA 11+ on injury prevention in amateur futsal players. Scand J Med Sci Sports 2020;30(8):1434-41 |
| 28 | Hilska et al.,2021 | Hilska M, Leppänen M, Vasankari T, et al. Neuromuscular Training Warm-up Prevents Acute Noncontact Lower Extremity Injuries in Children's Soccer: A Cluster Randomized Controlled Trial. Orthop J Sports Med 2021;9(4):23259671211005769 |
| 29 | Sommerfield et al., 2021 | Sommerfield LM, Whatman CS, Harrison CB, Maulder PS. The effects of a school-based injury prevention program on injury incidence in young females. J Sci Sport Exerc. 2021;3(1):47–55. |
| 30 | Obertinca et al., 2024 | Obërtinca R, Meha R, Hoxha I, Shabani B, Meyer T, Aus der Fünten K. Efficacy of a new injury prevention programme (FUNBALL) in young male football (soccer) players: a cluster-randomised controlled trial. Br J Sports Med 2024;58(10):548-55 |
