# Supplementary material for: Age-moderating effects and training characteristics of neuromuscular training for preventing knee injuries: a systematic review and meta-analysis
Source: Front Public Health. 2026 Aug 28;14:1822715. doi: 10.3389/fpubh.2026.1822715 (PMC13566741; doi:10.3389/fpubh.2026.1822715)
Supplement: Supplementary file 3 [file Supplementary_file_3.docx]

****Supplementary Appendix 3. Statistical results****

**1 Sensitivity analysis**

**1.1 Study-exclusion and cluster-adjustment sensitivity analyses**

**Table 1 Leave-one-out sensitivity analysis for knee injury outcomes**

| **Author (Year)** | **Log effect** | **95% CI** | **Back-transformed effect estimate**  **(95% CI)** | **p-value** |
| --- | --- | --- | --- | --- |
| Hewett et al., 1999 | -0.386 | -0.590 to -0.182 | 0.680 (0.554 to 0.834) | <0.001 |
| Wedderkopp et al., 1999 | -0.388 | -0.592 to -0.184 | 0.678 (0.553 to 0.832) | <0.001 |
| Heidt et al., 2000 | -0.400 | -0.608 to -0.193 | 0.670 (0.544 to 0.824) | <0.001 |
| Söderman M et al., 2000 | -0.422 | -0.626 to -0.218 | 0.656 (0.535 to 0.804) | <0.001 |
| Olsen et al., 2005 | -0.382 | -0.591 to -0.174 | 0.682 (0.554 to 0.840) | <0.001 |
| Petersen et al., 2005 | -0.399 | -0.606 to -0.191 | 0.671 (0.546 to 0.826) | <0.001 |
| Emery et al.,2007 | -0.424 | -0.631 to -0.217 | 0.654 (0.532 to 0.805) | <0.001 |
| Soligard et al., 2008 | -0.400 | -0.613 to -0.187 | 0.670 (0.542 to 0.829) | <0.001 |
| Steffen et al., 2008 | -0.427 | -0.637 to -0.218 | 0.652 (0.529 to 0.804) | <0.001 |
| Gilchrist et al., 2008 | -0.426 | -0.638 to -0.215 | 0.653 (0.528 to 0.807) | <0.001 |
| Emery & Meeuwisse, 2010 | -0.393 | -0.599 to -0.188 | 0.675 (0.549 to 0.829) | <0.001 |
| Kiani et al., 2010 | -0.375 | -0.576 to -0.175 | 0.687 (0.562 to 0.839) | <0.001 |
| LaBella et al., 2011 | -0.380 | -0.584 to -0.176 | 0.684 (0.558 to 0.839) | <0.001 |
| Longo et al., 2012 | -0.410 | -0.615 to -0.204 | 0.664 (0.541 to 0.815) | <0.001 |
| Waldén et al., 2012 | -0.422 | -0.636 to -0.209 | 0.656 (0.529 to 0.811) | <0.001 |
| van Beijsterveldt et al., 2012 | -0.396 | -0.608 to -0.184 | 0.673 (0.544 to 0.832) | <0.001 |
| Grooms et al., 2013 | -0.403 | -0.609 to -0.198 | 0.668 (0.544 to 0.820) | <0.001 |
| Owoeye et al.,2014 | -0.393 | -0.602 to -0.183 | 0.675 (0.548 to 0.833) | <0.001 |
| Finch et al., 2016 | -0.391 | -0.600 to -0.182 | 0.676 (0.549 to 0.834) | <0.001 |
| Silvers-Granelli et al., 2017 | -0.361 | -0.562 to -0.161 | 0.697 (0.570 to 0.851) | <0.001 |
| Bonato et al., 2018 | -0.361 | -0.553 to -0.169 | 0.697 (0.575 to 0.845) | <0.001 |
| Barber Foss et al., 2018 | -0.398 | -0.613 to -0.183 | 0.672 (0.542 to 0.833) | <0.001 |
| Slauterbeck et al., 2019 | -0.416 | -0.631 to -0.202 | 0.660 (0.532 to 0.817) | <0.001 |
| Bhat & Sreedhar, 2019 | -0.395 | -0.600 to -0.191 | 0.674 (0.549 to 0.826) | <0.001 |
| Yarsiasat et al., 2019 | -0.384 | -0.588 to -0.181 | 0.681 (0.555 to 0.834) | <0.001 |
| Akerlund et al., 2020 | -0.415 | -0.633 to -0.196 | 0.660 (0.531 to 0.822) | <0.001 |
| Lopes et al., 2020 | -0.403 | -0.610 to -0.196 | 0.668 (0.543 to 0.822) | <0.001 |
| Hilska et al.,2021 | -0.425 | -0.639 to -0.211 | 0.654 (0.528 to 0.810) | <0.001 |
| Sommerfield et al., 2021 | -0.438 | -0.616 to -0.260 | 0.645 (0.540 to 0.771) | <0.001 |
| Obertinca et al., 2024 | -0.407 | -0.621 to -0.194 | 0.666 (0.537 to 0.824) | <0.001 |
| Overall random-effects estimate | -0.401 | -0.604 to -0.198 | 0.670 (0.547 to 0.820) | <0.001 |

**Table 2 Age subgroup analysis after sensitivity analysis**

| **Analysis** | **Subgroup** | **k** | **Log effect** | **95% CI** | **Back-transformed effect estimate**  **(95% CI)** | **p-value** | **p for subgroup difference** | **I² (%)** | **p for heterogeneity** |
| --- | --- | --- | --- | --- | --- | --- | --- | --- | --- |
| All athletes | >18 years | 3 | -0.711 | -1.394 to -0.028 | 0.491 (0.248 to 0.972) | 0.041 | — | 0.0 | 0.816 |
|  | ≤18 years | 11 | -0.425 | -0.805 to -0.045 | 0.654 (0.447 to 0.956) | 0.029 | — | 68.2 | <0.001 |
|  | Overall | 14 | -0.493 | -0.825 to -0.160 | 0.611 (0.438 to 0.852) | 0.004 | 0.473 | 60.7 | 0.002 |

**Table 3 Sensitivity meta-regression analyses after excluding studies with unclear or unadjusted cluster adjustment**

| **Moderator** | **k** | **Coefficient β** | **SE** | **95% CI** | **Z-value** | **p-value** |
| --- | --- | --- | --- | --- | --- | --- |
| Mean age | 13 | -0.211 | 0.122 | -0.450 to 0.027 | -1.740 | 0.082 |
| Female proportion (%) | 14 | 0.001 | 0.004 | -0.008 to 0.009 | 0.210 | 0.835 |

**1.2 Effect-estimate-type sensitivity analyses**

**Table 4 Sensitivity analyses by effect-estimate type and age subgroup analyses restricted to risk-based estimates**

| **Analysis** | **Subgroup** | **k** | **Log effect** | **95% CI** | **Back-transformed effect estimate**  **(95% CI)** | **p-value** | **p for subgroup difference** | **I² (%)** | **p for heterogeneity** |
| --- | --- | --- | --- | --- | --- | --- | --- | --- | --- |
| Effect-estimate-type sensitivity analysis | Risk-based estimates:OR/RR | 24 | −0.375 | −0.632 to −0.117 | 0.687 (0.532–0.890) | 0.004 | 0.775 | 64.75 | <0.001 |
|  | Rate-based estimates:IRR/rate ratio | 6 | −0.425 | −0.655 to −0.195 | 0.654 (0.519–0.823) | <0.001 | — | 0.00 | 0.569 |
| Age subgroup analysis restricted to risk-based estimates | >18 years | 9 | −0.558 | −1.052 to −0.063 | 0.572 (0.349–0.939) | 0.027 | 0.357 | 64.67 | 0.004 |
|  | ≤18 years | 15 | −0.286 | −0.587 to 0.016 | 0.751 (0.556–1.016) | 0.064 | — | 63.73 | <0.001 |

**Note:** Risk-based estimates included event-count-based odds ratios (ORs) and reported risk ratios (RRs), whereas rate-based estimates included incidence rate ratios (IRRs) and rate ratios. Subgroup-specific estimates were calculated using random-effects models, and differences between subgroups were evaluated using mixed-effects models. Back-transformed estimates were obtained by exponentiating the pooled log estimates. Because the analyses included different but related ratio measures, results are described as pooled relative effect estimates rather than as pure pooled ORs, RRs, or IRRs. The p value for subgroup difference is shown only once for each analysis.

**Table 5 Sensitivity meta-regression analyses restricted to risk-based effect estimates**

| **Moderator** | **k** | **Coefficient β** | **SE** | **95% CI** | **Z-value** | **p-value** |
| --- | --- | --- | --- | --- | --- | --- |
| Mean age, years | 23 | −0.047 | 0.037 | −0.119 to 0.026 | −1.270 | 0.204 |
| Female proportion, % | 23 | 0.001 | 0.003 | −0.005 to 0.007 | 0.400 | 0.688 |

**Note:** The analyses were restricted to studies contributing risk-based effect estimates, including event-count-based odds ratios (ORs) and reported risk ratios (RRs). The dependent variable was the log-transformed relative effect estimate. β represents the change in the log-transformed effect estimate associated with a one-year increase in mean age or a one-percentage-point increase in female proportion. SE, standard error; CI, confidence interval.

**2 Publication bias**

**Table 6 Publication bias analyses for the overall pooled effect**

| **Method** | **Statistic / parameter** | **Result** |
| --- | --- | --- |
| Egger’s regression test | Intercept | -0.877 |
|  | Standard error | 0.574 |
|  | 95% CI | -2.054 to 0.299 |
|  | t-value | 1.527 |
|  | df | 28 |
|  | p-value, two-tailed | 0.138 |
| Begg and Mazumdar rank correlation | Kendall’s tau | -0.186 |
|  | p-value, two-tailed | 0.148 |
| Begg and Mazumdar rank correlation with continuity correction | Kendall’s tau | -0.184 |
|  | p-value, two-tailed | 0.154 |
| Duval and Tweedie trim-and-fill | Number of trimmed studies | 0 |
|  | Observed random-effects log estimate | -0.401 |
|  | Observed 95% CI | -0.604 to -0.198 |
|  | Adjusted random-effects log estimate | -0.401 |
|  | Adjusted 95% CI | -0.604 to -0.198 |
| Classic fail-safe N | Z-value for observed studies | -6.203 |
|  | Number of observed studies | 30 |
|  | Number of missing studies required to make p > 0.05 | 271 |

**Figure 1 Funnel plot for the overall sample**
